# The whole-genome assembly of an endangered Salicaceae species: *Chosenia arbutifolia* (Pall.) A. Skv

**DOI:** 10.1093/gigascience/giac109

**Published:** 2022-11-14

**Authors:** Xudong He, Yu Wang, Jinmin Lian, Jiwei Zheng, Jie Zhou, Jiang Li, Zhongyi Jiao, Yongchao Niu, Weiwei Wang, Jun Zhang, Baosong Wang, Qiang Zhuge

**Affiliations:** Willow Engineering Technology Research Center of National Forestry and Grassland Administration, Jiangsu Academy of Forestry, Nanjing 211153, China; Willow Nursery of the Jiangsu Provincial Platform for Conservation and Utilization of Agricultural Germplasm, Jiangsu Academy of Forestry, Nanjing 211153, China; Willow Engineering Technology Research Center of National Forestry and Grassland Administration, Jiangsu Academy of Forestry, Nanjing 211153, China; College of Biology and the Environment, Nanjing Forestry University, Nanjing 210037, China; Biozeron Shenzhen, Inc., Shenzhen 518000, China; Willow Engineering Technology Research Center of National Forestry and Grassland Administration, Jiangsu Academy of Forestry, Nanjing 211153, China; Willow Nursery of the Jiangsu Provincial Platform for Conservation and Utilization of Agricultural Germplasm, Jiangsu Academy of Forestry, Nanjing 211153, China; Willow Engineering Technology Research Center of National Forestry and Grassland Administration, Jiangsu Academy of Forestry, Nanjing 211153, China; Willow Nursery of the Jiangsu Provincial Platform for Conservation and Utilization of Agricultural Germplasm, Jiangsu Academy of Forestry, Nanjing 211153, China; Biozeron Shenzhen, Inc., Shenzhen 518000, China; Willow Engineering Technology Research Center of National Forestry and Grassland Administration, Jiangsu Academy of Forestry, Nanjing 211153, China; Willow Nursery of the Jiangsu Provincial Platform for Conservation and Utilization of Agricultural Germplasm, Jiangsu Academy of Forestry, Nanjing 211153, China; Biozeron Shenzhen, Inc., Shenzhen 518000, China; Willow Engineering Technology Research Center of National Forestry and Grassland Administration, Jiangsu Academy of Forestry, Nanjing 211153, China; Willow Nursery of the Jiangsu Provincial Platform for Conservation and Utilization of Agricultural Germplasm, Jiangsu Academy of Forestry, Nanjing 211153, China; Willow Engineering Technology Research Center of National Forestry and Grassland Administration, Jiangsu Academy of Forestry, Nanjing 211153, China; Willow Nursery of the Jiangsu Provincial Platform for Conservation and Utilization of Agricultural Germplasm, Jiangsu Academy of Forestry, Nanjing 211153, China; Willow Engineering Technology Research Center of National Forestry and Grassland Administration, Jiangsu Academy of Forestry, Nanjing 211153, China; Willow Nursery of the Jiangsu Provincial Platform for Conservation and Utilization of Agricultural Germplasm, Jiangsu Academy of Forestry, Nanjing 211153, China; College of Biology and the Environment, Nanjing Forestry University, Nanjing 210037, China

**Keywords:** *Chosenia arbutifolia*, genome assembly, phylogenetic relationship, genomic comparison

## Abstract

**Background:**

As a fast-growing tree species, *Chosenia arbutifolia* has a unique but controversial taxonomic status in the family Salicaceae. Despite its importance as an industrial material, in ecological protection, and in landscaping, *C. arbutifolia* is seriously endangered in Northeast China because of artificial destruction and its low reproductive capability.

**Results:**

To clarify its phylogenetic relationships with other Salicaceae species, we assembled a high-quality chromosome-level genome of *C. arbutifolia* using PacBio High-Fidelity reads and Hi-C sequencing data, with a total size of 338.93 Mb and contig N50 of 1.68 Mb. Repetitive sequences, which accounted for 42.34% of the assembly length, were identified. In total, 33,229 protein-coding genes and 11,474 small noncoding RNAs were predicted. Phylogenetic analysis suggested that *C. arbutifolia* and poplars diverged approximately 15.3 million years ago, and a large interchromosomal recombination between *C. arbutifolia* and other Salicaceae species was discovered.

**Conclusions:**

Our study provides insights into the genome architecture and systematic evolution of *C. arbutifolia*, as well as comprehensive information for germplasm protection and future functional genomic studies.

## Background

As a unique member of the family Salicaceae along with *Populus* and *Salix*, the genus *Chosenia* comprises only 1 species, *Chosenia arbutifolia* (Pall.) A. Skv. (NCBI:txid75699), according to the *Flora of China* [1]. Compared with poplars and willows, *C. arbutifolia* has several special morphological features, including an unusual leaf shape, extraordinary root system, and particular pistil, stamen, and bract structures [2]. Different from most insect-pollinated *Salix* species, *C. arbutifolia* is wind-pollinated and lacks nectary structures. Therefore, *C. arbutifolia* has been regarded as a transitional species between poplars and willows and treated as an independent genus by some authoritative botanists [3, 4]. However, ample molecular evidence demonstrated that *C. arbutifolia* has a close relationship with *Salix* species and should be considered a member of *Salix* [5]. To date, the taxonomic status of *C. arbutifolia* remains enigmatic and controversial.

*C. arbutifolia* is mostly distributed along the mountain river banks in Northeast China and in some areas of the Russian Far East, North Korea, and North Japan [2]. Even beyond the Arctic Circle, *C. arbutifolia* individuals are sporadically found [4]. Owing to its favorable characteristics of strong stress resistance, tremendous shape, and fast growth, *C. arbutifolia* is primarily applicable to industrial materials, ecological protection, and landscape planting. Unlike poplars and willows, *C. arbutifolia* is extremely difficult to propagate using twig cuttings, even when they originate from juvenile individuals [6]. In addition, the natural regeneration of *C. arbutifolia* by means of seed germination requires specific circumstances, including flowing water, an appropriate temperature, and sediment accumulation [4]. In the past decades, the growth area of *C. arbutifolia* has continuously decreased due to excessive deforestation. Furthermore, the species has a weak reproductive capability, resulting in a drastic decline in the natural populations of *C. arbutifolia*, and the species has been categorized as endangered in China.

Poplars and willows are expected to serve as novel model systems for genomic and genetic research in woody plants, mainly owing to their dioecism, short growth cycle, easy reproduction, and modest-sized genome [7]. The accomplishment of whole-genome sequencing of *Populus trichocarpa* in 2006 marked a new milestone and paved the way to a poplar genomic research field in the postgenomic era [8]. With the popularization of high-throughput sequencing technologies, numerous other *Populus* species and hybrids have been sequenced and assembled, including *Populuseuphratica* [9], *Populus pruinosa* [10], *Populus tremula* and *Populus tremuloides* [11], *Populus alba* [12], *P. alba* var. *pyramidalis* [13], *P. alba* × *P. tremula* var. *glandulosa* [14], and *P. ilicifolia* [15]. While similar work in the genus *Salix* is slightly lagging behind, an increasing number of whole-genome assemblies for the *Salix* species are being reported, including *Salix purpurea* (https://phytozome-next.jgi.doe.gov/info/Spurpurea_v5_1), *Salix brachista* [16], *Salix suchowensis* [17], *Salix viminalis* [18], *Salix matsudana* [19], and *Salix dunnii* [20]. Single-molecule real-time sequencing (SMRT), a third-generation sequencing technology, represents an optimal tool for whole-genome sequencing that overcomes various limits of short-read sequencing technologies and has been applied in some important woody plants, including *Liriodendron* [21], *Acer truncatum* [22], *Betula platyphylla* [23], *Paulownia fortune* [24], and *Taxus chinensis* var. *mairei* [25].

Despite its complex taxonomy, essential significance, and endangered status, available genetic and genomic information of *C. arbutifolia* is still scarce. Only a few studies that primarily focused on biological habits, propagation technology, population diversity and protection, phylogenetic analysis, transcriptome sequencing, and gene families are available and were reviewed by He et al. [26]. Here, with the aim to gain a deep insight into the genome architecture of *C. arbutifolia*, we assembled a chromosome-level and highly contiguous genome of *C. arbutifolia* using a combination of SMRT PacBio High-Fidelity (HiFi) reads, Illumina short-read sequencing, and the Hi-C chromosome conformation capture technology. We expected our work to provide substantial genomic resources of *C. arbutifolia* for future functional genomic research on Salicaceae.

## Methods

### Plant materials and nucleic acid extraction

Branches from a superior individual of *C. arbutifolia* were collected in the town of Manjiang (41°47′10.55″, 127°55′56.13″), Fusong County, Jilin Province, China. All branches were transported back to the laboratory of the Jiangsu Academy of Forestry for hydroponic cultivation until leaves had sprouted. A DNA extraction kit (DP305; Tiangen Biotech, Beijing, China) was used to isolate genomic DNA from young leaves of *C. arbutifolia*. An Omega Plant RNA Kit (Omega Bio-tek, Norcross, GA, USA) was used for total RNA extraction from the leaves of *C. arbutifolia*.

### Genome sequencing

According to the standard protocols (Pacific Biosciences, Menlo Park, CA, USA), genomic DNA was fragmented into ∼20-kb-long reads and used to prepare a PCR-free SMRT bell DNA library, which was sequenced using the circular consensus sequencing mode on the PacBio Sequel platform (RRID:SCR_017989). In addition, to generate PE150 short reads, short-insert libraries were constructed using the genomic DNA and then sequenced on the NovaSeq 6000 platform (Illumina NovaSeq 6000 Sequencing System, RRID:SCR_016387), following the manufacturer's instructions (Illumina, San Diego, CA, USA).

### Hi-C sequencing

The Dovetail Hi-C library preparation kit (Dovetail Genomics, Scotts Valley, CA, USA) was used for Hi-C library construction, according to the manufacturer's instructions. Briefly, formaldehyde was used to fix the nuclear chromatin. After extraction, the restriction enzyme, *Dpn*-II, was selected for digestion. Biotinylated nucleotides were filled and ligated to the sticky ends. After the revision of the crosslinks, free biotin was eliminated from the ligated fragments. The DNA was purified and sheared to ∼350 bp. Via streptavidin bead pulldown, biotinylated fragments were enriched and amplified by PCR for library construction. The library was sequenced on the Illumina NovaSeq platform.

### Genome assembly

The software DipAsm [27] was employed to construct contigs of *C. arbutifolia* using the PacBio HiFi reads to generate a haplotype-resolved assembly. The detailed parameters and settings used for DipAsm assembly are described in Supplementary Table S1. Then, the raw contigs were polished in 2 rounds based on the short reads generated by Illumina sequencing using the program Pilon v1.22 (RRID:SCR_014731) [28].

### Hi-C scaffolding

Hi-C technology was utilized to assist in the initial assembly to generate a chromosome-scale genome of *C. arbutifolia*. First, to filter the raw Hi-C reads, the program Hic-Pro v2.11.1 (RRID:SCR_017643) [29] was used to map the Illumina short reads onto the polished temporary genome with the default parameters. Then, invalid, nonligated, and self-ligated reads were discarded. Subsequently, the genomic contigs were clustered into potential chromosomal groups using the software Juicer v1.6.2 (RRID:SCR_017226) [30] and 3D de novo assembly v180114 (RRID:SCR_017227) [31]. Next, the contig orientation was validated using the assembly tool JuiceBox v1.11.8 (RRID:SCR_021172) [30] and the ambiguous fragments were removed manually. Finally, the completeness of the genome assembly was evaluated using the software BUSCO v5.2.1 (RRID:SCR_015008) [32]. The “CCCTAAA” telomeric repeat in the *C. arbutifolia* assembly was identified using Telomere Identification Toolkit.

### Characterization of repetitive sequences

The *C. arbutifolia* genome was screened for tandem and interspersed repeats. The software Tandem Repeats Finder v4.07 [33] was used to identify the tandem repeat contents. For the identification of interspersed repetitive sequences, a strategy combining *de novo* and given repeat searching was performed. The tools RepeatModeler v1.0.8 (RRID:SCR_015027) and LTR_FINDER v1.0.6 (RRID:SCR_015247) [34] were employed for the prediction of *de novo* repeat sequences. Then, RepeatMasker v4.0.7 (RRID:SCR_012954) was employed to screen the *C. arbutifolia* genome against the combined *de novo* transposable element library. RepeatMasker v4.0.7 and the Repbase database (RRID:SCR_021169) [35] were used to identify known transposable element repeats.

### Long terminal repeat insertion time estimation

The program LTR_FINDER v1.06 [34] was applied to detect long terminal repeats (LTRs) in the *C. arbutifolia* genome to estimate insertion times, with parameter settings “-D 15 000 -d 1000 -L 7000 -l 100 -p 20 -C -M 0.9.” Then, using the LTR_retriever (RRID:SCR_017623) pipeline, the results were integrated, and the false positives were removed from the primitive predictions. The insertion time was calculated as T = K/2r, where K and r represent the divergence rate and neutral mutation rate (r = 2.5 × 10^−9^), respectively [36].

### Genome annotation

The protein sequences of 7 plant genomes, including *Manihot esculenta, Linum usitatissimum, S. purpurea, P. trichocarpa, Ricinus communis, Jatropha curcas*, and *Arabidopsis thaliana*, were accessed from the NCBI and Phytozome database and mapped to the assembled genome of *C. arbutifolia* using the software genBlastA v1.0.4 (RRID:SCR_020951) [37]. Based on each genBlastA hit, the software GeneWise v2.4.1 (RRID:SCR_015054) [38] was employed to predict the exact gene structure. Three programs for *de novo* gene prediction, Augustus v3.2.1 (RRID:SCR_008417) [39], GlimmerHMM v3.0.4 (RRID:SCR_002654) [40], and SNAP v2006-07-28 (RRID:SCR_002127) [41], were applied to explore coding regions in the assembly of *C. arbutifolia*. The software HISAT2 v2.0.1 (RRID:SCR_015530) [42] was used to map RNA sequencing (RNA-seq) data to the chromosome-scaled *C. arbutifolia* assembly, and then StringTie v1.2.2 (RRID:SCR_016323) [43] was used to assemble the transcripts. The program TransDecoder v3.0.1 (RRID:SCR_017647) was conducted to identify the candidate coding regions. Using the above approaches, all predicted gene models were integrated by EVidenceModeler (RRID:SCR_014659) [44] into a nonredundant set of gene structures that were finally refined with the Program to Assemble Spliced Alignments (PASA) v2.3.3 (RRID:SCR_014656) [45]. The protein-coding genes were functionally annotated against 2 integrated SwissProt and TrEMBL databases using BLASTP (RRID:SCR_001010) [46] with E-value 1e-05. The software InterProScan v5.30 (RRID:SCR_005829) [47] was employed for protein domain annotation. For all genes, the BLAST Gene Ontology (GO) terms were extracted using InterProScan v5.30, and the pathways were assigned against the KEGG database (release 84.0) using BLAST (NCBI BLAST, RRID:SCR_004870).

### Noncoding RNA prediction

Noncoding RNAs, including 4 types of transfer RNAs (tRNAs), ribosomal RNAs (rRNAs), small nuclear RNAs (snRNAs), and micro-RNAs (miRNAs), were predicted. tRNAs and rRNAs were discovered using tRNAscan-SE v1.3.1 (RRID:SCR_010835) [48] and BLASTN v2.2.24 (RRID:SCR_001598, E-value 1e-5) via the alignment to template rRNA and tRNA sequences of *Oryza* and *Arabidopsis*, respectively. The snRNAs and miRNAs were screened from the Rfam database (RRID:SCR_007891) using INFERNAL v1.1.1 (RRID:SCR_011809).

### Gene family analysis

The OrthoMCL v2.0.9 (RRID:SCR_007839) [49] clustering program was run on the proteomes of *C. arbutifolia, S. purpurea, S. suchowensis, S. viminalis, S. brachista, P. euphratica, P. tremuloides, P. tremula, P. pruinosa, P. trichocarpa, P. alba*, and *R. communis*. A phylogenetic tree for these 12 species was constructed using the identified single-copy gene families. From each family, 4-fold degenerate sites were segregated and concatenated into 1 supergene. The phylogenetic tree was reestablished using the program MrBayes v3.1.2 (RRID:SCR_012067) with the model of GTR + gamma substitution. The program MCMCtree v4.4 in the PAML package (PRRID:SCR_014932) [50] was used to estimate the divergence times among the 12 species, with the JC69 nucleotide substitution model and an independent rates clock. The calibration divergence times between *S. purpurea* and *P. trichocarpa* (∼35.6 million years ago [MYA]), as well as *R. communis* and *P. trichocarpa* (∼80 MYA), were obtained from the TimeTree database (RRID:SCR_021162) [51]. Changes in gene family size within the phylogenetic tree were analyzed using CAFE v2.1 (RRID:SCR_005983) [52]. Positive selection genes in the *C. arbutifolia* genome were detected using the branch-site model incorporated in the PAML package (RRID:SCR_014932) [50] and a maximum likelihood ratio test based on the single-copy genes. *C. arbutifolia* and the other 11 species (except *R. communis*) were determined as foreground and background branches of the phylogenetic tree, respectively. GO enrichment was derived using Fisher's exact test followed by Benjamini–Hochberg adjustments, with the cutoff of *P* < 0.05. Whole-genome duplication (WGD) events were inferred based on the distribution of distance-transversion rate at 4-fold degenerate sites (4DTv) of paralogous gene pairs. The 4DTv transversion rates between all species pairs were calculated using an in-house Perl script.

### Synteny analysis

Syntenic regions between Salicaceae assemblies were based on homology searches carried out by conducting with MCScan (RRID:SCR_017650) (Python version) requiring at least 30 genes per block.

## Results and Discussion

### Genome assembly

**Figure 1:**
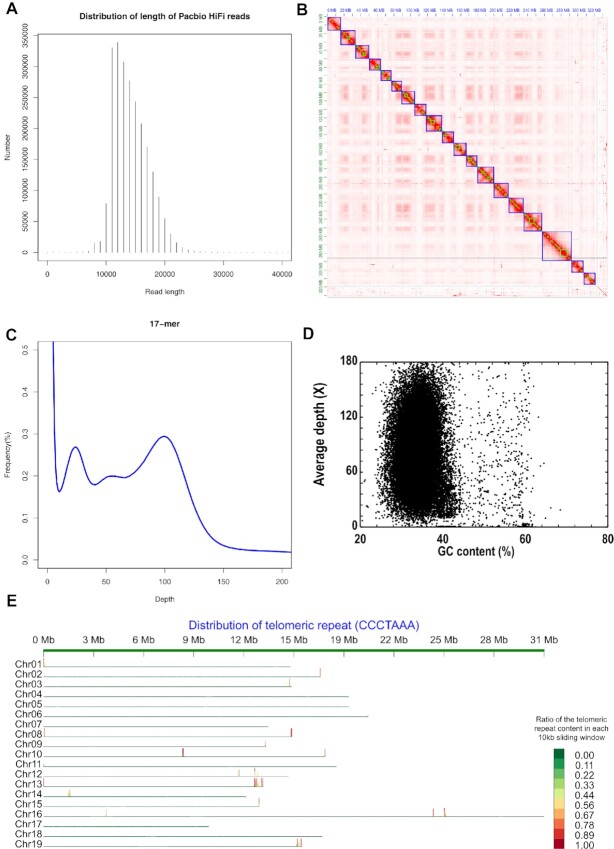
Genome sequencing and assembly of *C. arbutifolia*. (A) The length distribution of PacBio HiFi reads. x-axis: the length of HiFi reads; y-axis: the number of HiFi reads at a given length. (B) The Hi-C interaction heatmap for genome-wide analysis of chromatin interactions in the *C. arbutifolia* genome. (C) The 17-mer distribution of Illumina data. x-axis: the sequencing depth; y-axis: the proportion of a *k*-mer at a given sequencing depth. (D) The relationship between GC content and sequencing depths base on the alignment of PacBio HiFi data. (E) The distribution of “CCCTAAA” telomeric repeat in the 19 chromosomes of the *C. arbutifolia* assembly.

**Figure 2:**
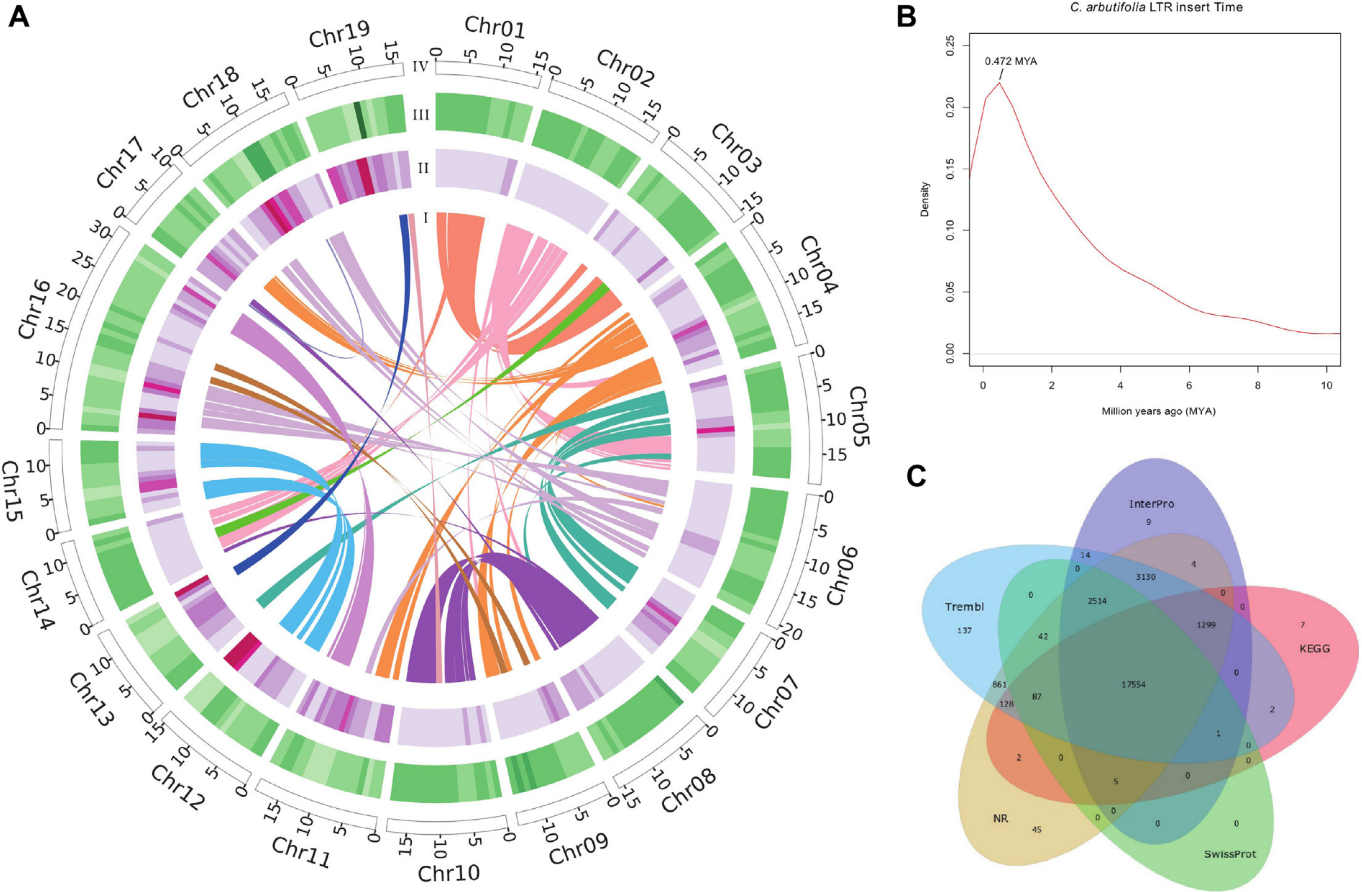
*C. arbutifolia* genome characeristics. (A) Genome circos plot. I: collinear regions within the *C. arbutifolia* assembly; II: percentage of transposable elements in 1-Mb sliding windows; III: gene density in 1-Mb sliding windows; IV: chromosome length in Mb. (B) Insertion times of LTR retrotransposons. (C) Venn diagram showing genes shared among different annotated datasets.

In total, 34.22 Gb with a ∼101× HiFi read coverage were generated through whole-genome sequencing of *C. arbutifolia* using the PacBio Sequel platform (Fig. 1A, Supplementary Table S2). The PacBio reads were assembled and polished with ∼111× Illumina paired-end reads (37.52 Gb, Supplementary Table S3), resulting in ∼1.68 Mb of contig N50 (Table 1). Subsequently, another 27.81 Gb Dovetail Hi-C data with a ∼82× depth were utilized to refine the genome assembly (Supplementary Table S3). Thus, a *C. arbutifolia* genome with a total size of 338.93 Mb was acquired, and 95.31% of the assembly sequence was assigned to 19 pseudochromosomes (Fig. 1B), which is close to the estimated genome size (323 Mb, Fig. 1C) and similar to those of *S. dunnii* [20] (328 Mb), *S. purpurea* (329.29 Mb, Table 1), and *S. brachista* [16] (339.58 Mb) but slightly smaller than those of *S.suchowensis* [17] (356.5Mb) and *S. viminalis* [18] (357.06 Mb). Compared with the genome sizes of *Populus* species, such as *P. trichocarpa* [8] (434.13 Mb), *P. euphratica* [9] (496.5 Mb), *P. pruinosa* [10] (479.3 Mb), *P. tremula* [11] (390 Mb), *P. tremuloides* [11] (378 Mb), *P. alba* [12] (415.99 Mb), *P. alba* var. *pyramidalis* [13] (464 Mb), and *P. ilicifolia* [15] (402 Mb), those of *C. arbutifolia* and *Salix* species are generally substantially smaller, which is consistent with early reports [17, 53]. The super-scaffold number, super-scaffold N50, and maximum super-scaffold length were 304, ∼16.46 Mb, and 31.95 Mb, respectively (Table 1). To evaluate the assembly quality of the *C. arbutifolia* genome, we mapped the next-generation sequencing short reads to the assembly, getting a mapping rate and coverage of 98.15% and 99.29%, respectively. The distribution of CG depth indicated there was no apparent contamination in the assembled sequences (Fig. 1D). A telomere unit “CCCTAAA” broadly discovered in plants was also detected in most assembled pseudochromosome sequences, except chromosomes 4, 5, 6, 7, 17, and 18 (Fig. 1E). In addition, 1,591 core genes were identified in the OrthoDB embryophyta database, accounting for 98.6% of the total 1,614 core genes, among which single-copy and duplicated genes represented 87.0% and 11.6%, respectively (Supplementary Table S4). The assembled *C. arbutifolia* genome and the features of different Salicaceae species are illustrated in Figure 2A and Table 1, respectively.

**Table 1:** Comparison of Salicaceae assemblies

Assembly feature	*C. arbutifolia*	*S. suchowensis*	*S. purpurea*	*P. trichocarpa*
Size (Mb)	338.93	356.5	329.29	434.13
No. of super-scaffolds	304	1,201	348	1,446
Contig N50 (bp)	1,682,645	263,908	5,083,238	552,806
Super-scaffold N50	16,460,042	16,776,717	14,688,223	19,465,461
Longest super-scaffold (Mb)	31.95	34.98	32.43	50.50
No. of protein coding genes	33,229	36,937	35,125	41,335
Complete BUSCOs (%)	98.6	97.3	98.4	98.8
LTR assembly index	10.86	17.06	13.82	8.15

### Repetitive sequence identification

Among the assembled genome sequences of *C. arbutifolia*, a total of ∼143.47 Mb (42.34%) repeat element sequences were screened, of which tandem and interspersed repeats accounted for 8.47% and 38.21%, respectively (Supplementary Table S5). Among the interspersed repeats, 3 types of repetitive elements, including class I (retrotransposons), class II (DNA transposons), and unclassified elements, representing 38.21% of the genome assembly, were identified (Supplementary Table S6). The LTR retrotransposons represented the most frequent among class I repetitive sequences, with Gypsy and Copia LTR retrotransposons accounting for 14.90% and 14.25%, respectively, whereas long and short interspersed nuclear elements represented approximately 3% of the genome size. The insertion time of LTR retrotransposons was predicted by detecting the sequence divergence at both ends of impact LTRs. As shown in Fig. 2B, a surge of retrotransposon amplification was detected in *C. arbutifolia* approximately 0.472 MYA, indicating an expansion event in the recent period of genome evolution.

### Gene annotation

Through a combined prediction strategy of *ab initio*, homologous protein, and transcriptome, 33,229 protein-coding genes were predicted (Supplementary Table S6). Of these, 31,618 (95.15%) were successfully annotated in diverse databases, including NCBI nr, Swissprot, KEGG, TrEMBL, and InterPro, whereas the remaining 1,611 (4.85%) genes had no significant correspondence with sequences in public databases (Supplementary Table S7, Fig. 2C). In addition, the complete BUSCO of the predicted proteins is about 97.3% (Supplementary Table S8), indicating the high quality of the annotated genes. The reason for the overall smaller genomes of the *Salix* species has been suggested to be the faster evolution speed of willows, which reduces the predicted gene number [17, 53]. However, we found that there is no linear correspondence between the genome size and the number of predicted genes in the Salicaceae species. For example, 36,490 genes have been identified in *S. viminalis* [18] in an assembled genome of 357.06 Mb. The *S. viminalis* genome is smaller in size but harbors a larger number of genes than those of *P. pruinosa* [10] (35,131 genes), *P. alba* [12] (32,963 genes), and *P. ilicifolia* [15] (33,684 genes). *S. brachista* [16] has a slightly larger genome (339.58 Mb) but smaller number of predicted genes (30,209) than *C. arbutifolia* (338.93 Mb; 33,229 genes) and *S. purpurea* (329.29 Mb; 35,125 genes). Undoubtedly, the efficiency of genome assembly and the strategy used for gene mining are essential factors affecting the numbers of predicted genes in different species. The mean length of the predicted protein-coding genes was 3,156 bp, with 5.02 exons per gene, and the average lengths of exons and introns were 233 bp and 446 bp, respectively (Supplementary Table S9). Noncoding RNAs in the *C. arbutifolia* genome were explored and annotated, and they comprised 239 miRNAs, 697 tRNAs, 10,043 rRNAs, and 495 snRNAs (Supplementary Table S10).

### Phylogenetic relationship analysis

**Figure 3:**
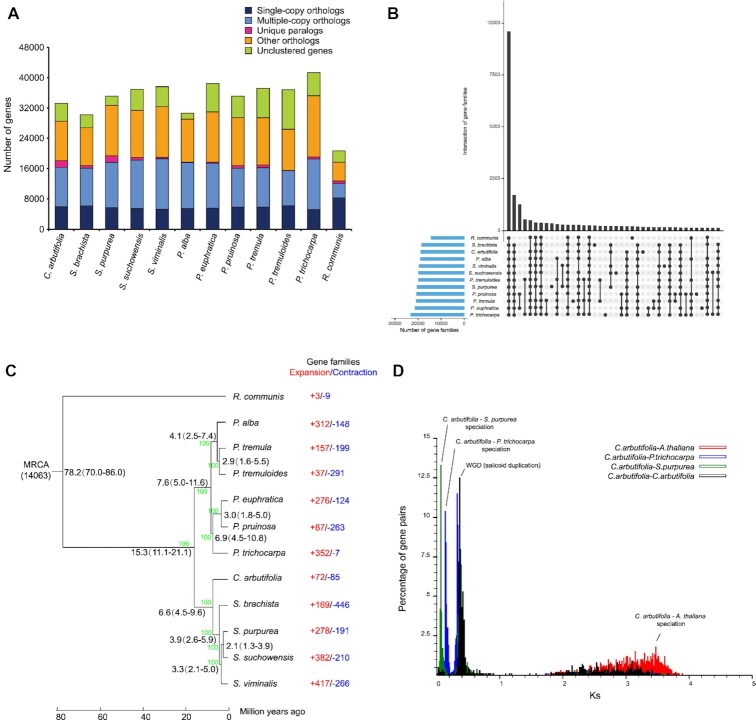
Genome comparison of different Malpighiales species. (A) Protein orthology comparison in genomes of the indicated 12 species. (B) Intersections of gene families among the 12 species. Rows and columns represent gene families and intersections, respectively. Black and gray circles indicate the existence or absence of a given intersection. Vertical black lines connecting black circles in each column represent the column-based relationship. The bar chart located at the top of the matrix indicates the intersection size. The horizontal bar chart on the left side of the matrix indicates the size of gene family. (C) Phylogenetic tree of the 12 species. Numbers (black) on nodes indicate the differentiation time, and error ranges are shown in parentheses. (D) Genome duplication in the *C. arbutifolia* genome revealed by 4DTv analysis.

The protein-coding genes of 11 Malpighiales species, including *S. purpurea, S. suchowensis, S. viminalis, S. brachista, P. trichocarpa, P. tremuloides, P. tremula, P. pruinosa, P. euphratica, P. alba*, and *R. communis*, were collected from relevant databases and clustered into 30,618 gene families together with the protein-coding genes of *C. arbutifolia*(Supplementary Table S11, Fig. 3A). The analysis of gene family intersection exhibited that 11,308 gene families were shared by the 11 Salicaceae species but not *R. communis* (Fig. 3B). For *C. arbutifolia*, 28,512 genes were assigned to 18,729 genes families, of which 184 families, containing 1,750 genes in total, were specific when compared with the 11 other Malpighiales species (Supplementary Table S11). These genes were significantly enriched in the GO terms “DNA binding,” “ribonucleoside binding,” and “DNA-directed 5′-3′ RNA polymerase activity” with a false discovery rate <0.05 (Supplementary Table S12).

A phylogenetic tree was constructed for the 12 Malpighiales species, considering *R. communis* as an outgroup (Fig. 3C). The divergence time between *Chosenia* and *Populus* was assessed to be around 15.3 MYA, and *C. arbutifolia* was separated from the 4 *Salix* species around ∼6.6 MYA, indicating that *C. arbutifolia* was the first species to differentiate from *Populus* and may be a transitional species between poplars and willows. It preserved some poplar characteristics, such as the haploid number (*n* = 19, most of the tree species in *Salix* are polyploid), wind pollination, and absence of glands. In addition, previous reports on *S. brachista* [16] and *S. dunnii* [20] demonstrate exactly the same relationships and similar divergence times among the abovementioned species, indicating that *S. suchowensis* may have evolved substantially further than other *Salix* species owing to a stronger purifying selection [17, 53]. However, the family Salicaceae comprises more than 600 species worldwide, and limited available genome data of Salicaceae species were analyzed in this study. Thus, to completely clarify the phylogenetic history of this family, more species should be added in the future.

Compared with the most recent common ancestor (MRCA) of *Chosenia* and *Salix, C. arbutifolia* showed 72 and 85 expansion and contraction events of each gene family, respectively (Fig. 3C). The results of GO enrichment analysis revealed that among the expanded genes, 29 genes were associated with “heme binding” and “oxidation reduction process,” and 22 genes were involved in “iron ion binding” (Supplementary Table S13). Among the contracted genes, 93 genes were related to “ATP binding,” and 88 genes were responsible for “protein kinase activity” and “protein phosphorylation” (Supplementary Table S13). Positive selection genes (PSGs) were detected using single-copy gene sets of the 12 species. In *C. arbutifolia*, a total of 89 PSGs were detected, of which 6 and 5 PSGs were enriched in the GO terms of “integral component of membrane” and “catalytic activity,” respectively, whereas 3 PSGs were related to both “ATP binding” and “nucleic acid binding” (Supplementary Table S14).

### Whole-genome duplication analysis

WGD events were deduced by examining distributions of synonymous substitutions per site (Ks) within the *C. arbutifolia* genome. After the speciation between *C. arbutifolia* and *A. thaliana* (Ks = 3.47), a common salicoid WGD event occurred (Ks = 0.36). The divergence between *C. arbutifolia* and *P. trichocarpa* emerged at the peak of Ks ∼0.13, followed by *C. arbutifolia* and *S. purpurea* (Ks = 0.06), which is inconsistent with the results of phylogenetic analysis (Fig. 3D). After the differentiation of the Salicaceae species, there was no obvious evidence of a *C. arbutifolia*–specific WGD.

### Genome collinearity analysis

**Figure 4:**
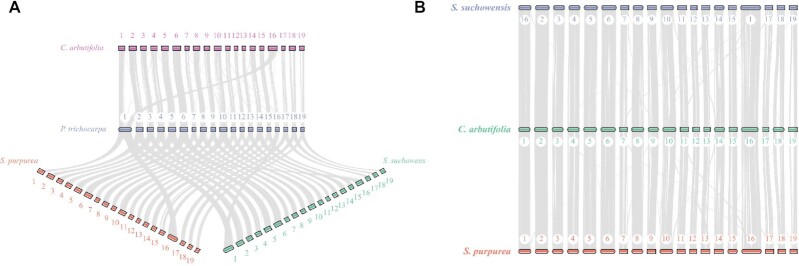
Synteny analysis. (A) Synteny analysis of *C. arbutifolia, S. purpurea, S. suchowensis*, and *P. trichocarpa*. (B) Synteny analysis of *C. arbutifolia, S. purpurea*, and *S. suchowensis*. Macrosynteny connecting blocks of >30 one-to-one gene pairs are shown.

The genome collinearity among *C. arbutifolia, S. purpurea, S. suchowensis*, and *P. trichocarpa* was analyzed. The syntenic regions showed that most chromosomes were highly conserved among the Salicaceae species, except for a large interchromosomal recombination between chromosomes 1 and 16 (Fig. 4A). Compared with *P. trichocarpa*, we have identified 2,135, 3,351, and 2,829 genes in the whole-genome interchromosomal-recombination regions for *C. arbutifolia, S. purpurea*, and *S. suchowensis*, respectively (Supplementary Table S15, Supplementary Table S16). Furthermore, the whole chromosomes of *C. arbutifolia* and the 2 *Salix* species were highly collinear (Fig. 4B). Together, these results indicated that main chromosomal fissions and fusions have occurred during the evolution of Salicaceae, resulting in a genera divergence of Salicaceae. Like in other *Salix* species, such as *S. brachista* [16], *S. suchowensis* [17, 53], and *S. dunnii* [20], most of the chromosomes of *C. arbutifolia* were highly conserved with *P. trichocarpa*, except for chromosomes 1 and 16, in which a large interchromosomal recombination was discovered (Fig. 4A). It has been reported that the chromosomal fusions and fissions that emerged in *Populus* after a lineage-specific salicoid duplication gave rise to the divergence of the 2 genera, *Populus* and *Salix* [54]. Nevertheless, recombination modes are quite different between *S. suchowensis* and other *Salix* species. Chromosome 16 of *S. suchowensis* entirely originated from a partial chromosome 1 of *P. trichocarpa*, and chromosome 1 of *S. suchowensis* comprised the remaining part of chromosome 1 and the entire chromosome 16 of *P. trichocarpa* [17]. However, in our study, chromosome 16 of *C. arbutifolia* was fused with a partial chromosome 1 and the entire chromosome 16 of *P. trichocarpa*, and chromosome 1 of *C. arbutifolia* comprised the remaining part of *P. trichocarpa* chromosome 1. This difference was confirmed by collinearity analysis (Fig. 4B), and the same phenomenon was also detected in *S. brachista* [16] and *S. dunnii* [20].

## Conclusions

Although multiple genomes of *Populus* and *Salix* species have been reported, we sequenced and assembled a genome of the taxonomically difficult species *C. arbutifolia* that belongs to the monotypic genus *Chosenia* for the first time by using PacBio HiFi reads, Hi-C chromatin contact maps, and Illumina short reads. As a significant supplementary for the family Salicaceae, the assembled genome shed a deep insight into the genomic architecture of *C. arbutifolia* and revealed the systematic evolution and phylogenetic relationships with other Salicaceae species. Given the limited genomic resources in the public databases, it is worthwhile to take full advantage of more available genomic information for further studies. Overall, our results lay a solid foundation for genetic and genomic research on Salicaceae species in the future. All supporting data and materials are available in the GigaScience GigaDB database [55].

## Additional Files

**Supplementary Table S1**. The parameters and settings used for DipAsm assembly.

**Supplementary Table S2**. Statistics of PacBio HiFi data.

**Supplementary Table S3**. Statistics of Illumina data.

**Supplementary Table S4**. Statistics of the *C. arbutifolia* assembly gene space with the 1,440 BUSCO embryophyta gene set.

**Supplementary Table S5**. General statistics of the repeats in the *C. arbutifolia* genome.

**Supplementary Table S6**. Interspersed repeat (TE) content in the assembled *C. arbutifolia* genome.

**Supplementary Table S7**. Functional annotation of the predicted genes for *C. arbutifolia*.

**Supplementary Table S8**. Statistics of the predicted protein-coding genes in different species.

**Supplementary Table S9**. Statistic of the predicted proteins with the 1,614 BUSCO embryophyta gene set.

**Supplementary Table S10**. Noncoding RNAs in the *C. arbutifolia* genome.

**Supplementary Table S11**. Statistics of gene families of the 12 Malpighiales species.

**Supplementary Table S12**. GO enrichment of the specific genes in *C. arbutifolia*.

**Supplementary Table S13**. GO enrichment of expanded and contracted genes in *C. arbutifolia*.

**Supplementary Table S14**. GO enrichment of positive selection genes in *C. arbutifolia*.

**Supplementary Table S15**. The interchromosomal-recombination blocks between Salicaceae species.

**Supplementary Table S16**. Statistic of the gene numbers in interchromosomal-recombination regions between Salicaceae species.

giac109_GIGA-D-22-00145_Original_Submission

giac109_GIGA-D-22-00145_Revision_1

giac109_Response_to_Reviewer_Comments_Original_Submission

giac109_Reviewer_1_Report_Original_Submission Jian-Feng Mao, Ph.D. -- 7/18/2022 Reviewed

giac109_Reviewer_2_Report_Original_Submission Bartosz Ulaszewski -- 7/31/2022 Reviewed

giac109_Reviewer_3_Report_Original_Submission Tao Ma -- 8/8/2022 Reviewed

giac109_Reviewer_3_Report_Revision_1 Tao Ma -- 9/30/2022 Reviewed

giac109_Supplemental_Tables

## Data Availability

The genome assembly and all the sequencing data have been deposited in the GenBank database under the accession number PRJNA788330. All supporting data and materials are available in the *GigaScience* GigaDB database [55].

## Abbreviations

4DTv: 4-fold degenerate sites; BLAST: Basic Local Alignment Search Tool; bp: base pair; BUSCO: Benchmarking Universal Single-Copy Orthologs; Gb: gigabase; GO: Gene Ontology; HiFi: High-Fidelity; kb: kilobase; KEGG: Kyoto Encyclopedia of Genes and Genomes; LTR: long terminal repeat; miRNAs: micro-RNAs; MRCA: most recent common ancestor; MYA: million years ago; PSGs: positive selection genes; RLKs: receptor-like kinases; rRNAs: ribosomal RNAs; RNA-seq: RNA sequencing; PASA: Program to Assemble Spliced Alignments; SMRT: Single-Molecule Real-time Sequencing; snRNAs: small nuclear RNAs; tRNAs: transfer RNAs; WGD: whole-genome duplication.

## Funding

This work was financially supported by the National Natural Science Foundation of China (Grant No. 31670662) and the Independent Scientific Research Project of Jiangsu Academy of Forestry (Grant No. ZZKY202101).

## Competing Interests

The authors declare that they have no conflict of interest.

## Authors' Contributions

XDH and QZ conceived and designed the experiments. XDH wrote and revised the manuscript. YW, JML, JWZ, JZ, JL, ZYJ, and YCN analyzed the data. BSW and WWW collected the samples. JZ processed the data. All authors have read and approved the final manuscript.
