# Supplementary material for: The whole-genome assembly of an endangered Salicaceae species: Chosenia arbutifolia (Pall.) A. Skv
Source: Gigascience. 2022 Nov 14;11:giac109. doi: 10.1093/gigascience/giac109 (PMC9661892; doi:10.1093/gigascience/giac109)
Supplement: giac109_GIGA-D-22-00145_Original_Submission [file giac109_giga-d-22-00145_original_submission.pdf]

GigaScience

The whole-genome assembly of an endangered Salicaceae species: Chosenia arbutifolia (Pall.) A. Skv.

--Manuscript Draft--

**Manuscript Number:**	GIGA-D-22-00145
**Full Title:**	The whole-genome assembly of an endangered Salicaceae species: Chosenia arbutifolia (Pall.) A. Skv.
**Article Type:**	Data Note
**Funding Information:**	National Natural Science Foundation of China (31670662) — Dr. Xudong He<br>Independent Scientific Research Project of Jiangsu Academy of Forestry (ZZKY202101) — Prof. Baosong Wang
**Abstract:**	Background<br><br>As a fast-growing tree species, Chosenia arbutifolia has a unique, but controversial taxonomic status in the family Salicaceae. Despite its importance as an industrial material, in ecological protection, and in landscaping, C. arbutifolia is seriously endangered in Northeast China because of artificial destruction and its low reproductive capability.<br><br>Results<br><br>To clarify its phylogenetic relationships with other Salicaceae species, we assembled a high-quality chromosome-level genome of C. arbutifoli a using PacBio HiFi reads and Hi-C sequencing data, with a total size of 338.93 Mb and contig N50 of 1.68 Mb. Repetitive sequences, which accounted for 42.34% of the assembly length, were identified. In total, 33,229 protein-coding genes and 11,474 small ncRNAs were predicted. Phylogenetic analysis suggested that C. arbutifolia and poplars diverged approximately 15.3 million years ago, and a large interchromosomal recombination between C. arbutifolia and other Salicaceae species was discovered.<br><br>Conclusions<br><br>Our study provides insights into the genome architecture and systematic evolution of C. arbutifolia , as well as comprehensive information for germplasm protection and future functional genomic studies.
**Corresponding Author:**	Xudong He<br>Jiangsu Academy of Forestry<br>Nanjing, Jiangsu CHINA
**Corresponding Author Secondary Information:**	
**Corresponding Author's Institution:**	Jiangsu Academy of Forestry
**Corresponding Author's Secondary Institution:**	
**First Author:**	Xudong He
**First Author Secondary Information:**	
**Order of Authors:**	Xudong He<br>Yu Wang<br>Jinmin Lian<br>Jiwei Zheng<br>Jie Zhou<br>Jiang Li

Powered by Editorial Manager® and ProduXion Manager® from Aries Systems Corporation

	Zhongyi Jiao
	Yongchao Niu
	Weiwei Wang
	Jun Zhang
	Baosong Wang
	Qiang Zhuge
**Order of Authors Secondary Information:**	
**Additional Information:**	
**Question**	**Response**
Are you submitting this manuscript to a special series or article collection?	No
**Experimental design and statistics**<br><br>Full details of the experimental design and statistical methods used should be given in the Methods section, as detailed in our Minimum Standards Reporting Checklist. Information essential to interpreting the data presented should be made available in the figure legends.<br><br>Have you included all the information requested in your manuscript?	Yes
**Resources**<br><br>A description of all resources used, including antibodies, cell lines, animals and software tools, with enough information to allow them to be uniquely identified, should be included in the Methods section. Authors are strongly encouraged to cite Research Resource Identifiers (RRIDs) for antibodies, model organisms and tools, where possible.<br><br>Have you included the information requested as detailed in our Minimum Standards Reporting Checklist?	Yes
**Availability of data and materials**<br><br>All datasets and code on which the	Yes

conclusions of the paper rely must be either included in your submission or deposited in publicly available repositories (where available and ethically appropriate), referencing such data using a unique identifier in the references and in the "Availability of Data and Materials" section of your manuscript.<br><br>Have you have met the above requirement as detailed in our Minimum Standards Reporting Checklist?	

Powered by Editorial Manager® and ProduXion Manager® from Aries Systems Corporation

Manuscript
Click here to access/download;Manuscript;Manuscript for giga.doc

DATANOTE

The whole-genome assembly of an endangered Salicaceae species: *Chosenia arbutifolia* (Pall.) A. Skv.

Xudong He[1,2,†*], Yu Wang[1,3,†], Jinmin Lian[4,†], Jiwei Zheng[1,2], Jie Zhou[1,2], Jiang Li[4], Zhongyi Jiao[1,2], Yongchao Niu[4], Weiwei Wang[1,2], Jun Zhang[1,2], Baosong Wang[1,2], Qiang Zhuge[3]

[1] Willow Engineering Technology Research Center of National Forestry and Grassland Administration, Jiangsu Academy of Forestry, Nanjing 211153, China

[2] Willow Nursery of the Jiangsu Provincial Platform for Conservation and Utilization of Agricultural Germplasm, Jiangsu Academy of Forestry, Nanjing 211153, China

[3] College of Biology and the Environment, Nanjing Forestry University, Nanjing 210037, China

[4] Biozeron Shenzhen, Inc., Shenzhen 518000, China

[*] Corresponding author: Xudong He

E-mail: hxd_519@163.com

Tel: +86 25 52743830

Fax: +86 25 52741620

[†] These authors contributed equally to this work.

Abstract

Background As a fast-growing tree species, *Chosenia arbutifolia* has a unique, but controversial taxonomic status in the family Salicaceae. Despite its importance as an industrial material, in ecological protection, and in landscaping, *C. arbutifolia* is seriously endangered in Northeast China because of artificial destruction and its low reproductive capability.

Results To clarify its phylogenetic relationships with other Salicaceae species, we assembled a high-quality chromosome-level genome of *C. arbutifoli*a using PacBio HiFi reads and Hi-C sequencing data, with a total size of 338.93 Mb and contig N50 of 1.68 Mb. Repetitive sequences, which accounted for 42.34% of the assembly length, were identified. In total, 33,229 protein-coding genes and 11,474 small ncRNAs were predicted. Phylogenetic analysis suggested that *C. arbutifolia* and poplars diverged approximately 15.3 million years ago, and a large interchromosomal recombination between *C. arbutifolia* and other Salicaceae species was discovered.

Conclusions Our study provides insights into the genome architecture and systematic evolution of *C. arbutifolia*, as well as comprehensive information for germplasm protection and future functional genomic studies.

Keywords *Chosenia arbutifolia*, genome assembly, phylogenetic relationship, genomic comparison

Data Description

Background

As a unique member of the family Salicaceae along with *Populus* and *Salix*, the genus *Chosenia* comprises only one species, *C. arbutifolia* (Pall) A. Skv., according to the Flora of China [1]. Compared with poplars and willows, *C. arbutifolia* has several special morphological features, including an unusual leaf shape, extraordinary root system, and particular pistil, stamen, and bract structures [2]. Different from willows, *C. arbutifolia* is wind-pollinated and lacks nectary structures. Therefore, *C. arbutifolia* has been regarded a transitional species between poplars and willows and treated as an independent genus by some authoritative botanists [3, 4]. However, ample molecular evidence demonstrated that *C. arbutifolia* has a close relationship with *Salix* species and should be considered a member of *Salix* [5]. To date, the taxonomic status of *C. arbutifolia* remains enigmatic and controversial.

C. arbutifolia is mostly distributed along the mountain river banks in Northeast China, and in some areas of the Russian Far East, North Korea, and North Japan [2]. Even beyond the Arctic Circle, *C. arbutifolia* individuals are sporadically found [4]. Owing to its favorable characteristics of strong stress resistance, tremendous shape, and fast growth, *C. arbutifolia* is primarily applicable to industrial materials, ecological protection, and landscape planting. Unlike poplars and willows, *C. arbutifolia* is extremely difficult to propagate using twig cuttings, even when they originate from juvenile individuals [6]. In addition, the natural regeneration of *C. arbutifolia* by means of seed germination requires specific circumstances, including flowing water, an appropriate temperature, and sediment accumulation [4]. In the past decades, the growth area of *C. arbutifolia* has continuously decreased due to excessive deforestation. Furthermore, the species has a weak reproductive capability, resulting in a drastical decline in the natural populations of *C. arbutifolia* and the species has been categorized as an endangered in China.

Poplars and willows are expected to serve as novel model systems for genomic and genetic research in woody plants, mainly owing to their dioecism, short growth cycle, easy reproduction, and modest-sized genome [7]. The accomplishment of whole-genome sequencing of *P. trichocarpa* in 2006 marked a new milestone and paved the way

to a poplar genomic research field in the post-genomic era [8]. With the popularization of high-throughput sequencing technologies, numerous other *Populus* species and hybrids have been sequenced and assembled, including *P. euphratica* [9], *P. pruinosa* [10], *P. tremula* and *P. tremuloides* [11], *P. alba* [12], *P. alba* var. *pyramidalis* [13], *P. alba* × *P. tremula* var. *glandulosa* [14], and *P. ilicifolia* [15]. While similar work in the genus *Salix* is slightly lagging behind, an increasing number of whole genome assemblies for the *Salix* species are being reported, including *S. purpurea* (https://phytozome-next.jgi.doe.gov/info/Spurpurea_v5_1), *S. brachista* [16], *S. suchowensis* [17], *S. viminalis* [18], *S. matsudana* [19], and *S. dunnii* [20]. Single-molecule real-time sequencing (SMRT), a third-generation sequencing technology, represents an optimal tool for whole-genome sequencing that overcomes various limits of short-reads sequencing technologies, and has been applied in some important woody plants, including *Liriodendron* [21], *Acer truncatum* [22], *Betula platyphylla* [23], *Paulownia fortune* [24], and *Taxus chinensis* var. *mairei* [25].

Despite its complex taxonomy, essential significance, and endangered status, available genetic and genomic information of *C. arbutifolia* are still scarce. Only a few studies that primarily focused on biological habits, propagation technology, population diversity and protection, phylogenetic analysis, transcriptome sequencing, and gene families are available and were reviewed by He et al. [26]. Here, with the aim to gain a deep insight into the genome architecture of *C. arbutifolia*, we assembled a chromosome-level and highly contiguous genome of *C. arbutifolia* using a combination of SMRT PacBio High-Fidelity (HiFi) reads, Illumina short-read sequencing, and the Hi-C chromosome conformation capture technology. We expected our work to provide substantial genomic resources of *C. arbutifolia* for future functional genomic research on Salicaceae.

Methods

Plant materials and nucleic acid extraction

Branches from superior individuals of *C. arbutifolia* and *S. suchowensis* were collected in the town of Manjiang

(41°47′10.55″, 127°55′56.13″), Fusong County, Jilin Province, China, and in the willow nursery of the Jiangsu Academy of Forestry, Nanjing, Jiangsu Province, China. All branches were transported back to the laboratory for hydroponic cultivation until leaves and roots had sprouted (no roots for *C. arbutifolia*). A DNA extraction kit (DP305, Tiangen Biotech, Beijing, China) was used to isolate genomic DNA from young leaves of *C. arbutifolia*. An Omega Plant RNA Kit (Omega Bio-tek, Norcross, GA, USA) was used for total RNA extraction from the leaves of *C. arbutifolia* and *S. suchowensis* and the ARs of *S. suchowensis*.

Genome sequencing

According to the standard protocols (Pacific Biosciences, Menlo Park, CA, USA), genomic DNA was fragmented into ~20-kb long reads and used to prepare a PCR-free SMRT bell DNA library, which was sequenced using the circular consensus sequencing mode on the PacBio Sequel platform (RRID:SCR_017989). In addition, to generate PE150 short reads, short-insert libraries were constructed using the genomic DNA and then sequenced on the NovaSeq 6000 platform (RRID:SCR_016387), following the manufacturer's instructions (Illumina, San Diego, CA, USA).

Hi-C sequencing

The Dovetail Hi-C library preparation kit (Dovetail Genomics, Scotts Vally, CA, USA) was used for Hi-C library construction, according to the manufacturer's instructions. Briefly, formaldehyde was used to fix the nuclear chromatin. After extraction, the restriction enzyme, *Dpn*-II was selected for digestion. Biotinylated nucleotides were filled and ligated to the sticky ends. After revision of the crosslinks, free biotin was eliminated from the ligated fragments. The DNA was purified and sheared to ~350 bp. Via streptavidin bead pulldown, biotinylated fragments were enriched and amplified by PCR for library construction. The library was sequenced on the Illumina NovaSeq platform (RRID:SCR_016387).

Genome assembly

The software DipAsm [27] was employed to construct contigs of *C. arbutifolia* using the Pacbio HiFi reads to generate a haplotype-resolved assembly. Then, the raw contigs were polished in two rounds based on the short reads generated by Illumina sequencing using the program Pilon v1.22 (RRID:SCR_014731) [28].

Hi-C scaffolding

Hi-C technology was utilized to assist the initial assembly to generate a chromosome-scale genome of *C. arbutifolia*. First, to filter the raw Hi-C reads, the program Hic-Pro v2.11.1 (RRID:SCR_017643) [29] was used to map the Illumina short reads onto the polished temporary genome with the default parameters. Then, invalid, non-ligated, and self-ligated reads were discarded. Subsequently, the genomic contigs were clustered into potential chromosomal groups using the software Juicer v1.6.2 (RRID:SCR_017226) [30] and 3d-DNA v180114 (RRID:SCR_017227) [31]. Next, the contig orientation was validated using the assembly tool JuiceBox v1.11.8 (RRID:SCR_021172) [30] and the ambiguous fragments were removed manually. Finally, the sequence integrity of the assembled genome was evaluated using the software BUSCO v5.2.1 (RRID:SCR_015008) [32].

Characterization of repetitive sequences

The *C. arbutifolia* genome was screened for tandem and interspersed repeats. The software Tandem Repeats Finder v4.07 (RRID:SCR_022193) [33] was used to identify the tandem repeat contents. For the identification of interspersed repetitive sequences, a strategy combining *de novo* and given repeat searching was performed. The tools RepeatModeler v1.0.8 (RRID:SCR_015027, https://github.com/Dfam-consortium/RepeatModeler) and LTR_FINDER v1.0.6 (RRID:SCR_015247) [34] were employed for the prediction of *de novo* repeat sequences. Then, RepeatMasker v4.0.7 (RRID:SCR_012954, https://github.com/rmhubley/RepeatMasker) was employed to screen the *C. arbutifolia* genome against the combined *de novo* transposable element library. RepeatMasker v4.0.7

and the Repbase database (RRID:SCR_021169) [35] were used to identify known transposable element repeats.

LTR insertion time estimation

The program LTR_FINDER v1.06 (RRID:SCR_015247) [34] was applied to detect LTRs in the *C. arbutifolia* genome to estimate insertion times, with parameter settings '-D 15000 -d 1000 -L 7000 -l 100 -p 20 -C -M 0.9'. Then, using the LTR_retriever (RRID:SCR_017623) pipeline, the results were integrated, and the false positives were removed from the primitive predictions. The insertion time was calculated as $T = K / 2r$, where K and r represent the divergence rate and neutral mutation rate ($r = 2.5 \times 10^{-9}$), respectively.

Genome annotation

The protein sequences of seven plant genomes, including *Manihot esculenta*, *Linum usitatissimum*, *S. purpurea*, *P. trichocarpa*, *R. communis*, *Jatropha curcas*, and *Arabidopsis thaliana*, were accessed from the NCBI and Phytozome database and mapped to the assembled genome of *C. arbutifolia* using the software genBlastA v1.0.4 (RRID:SCR_020951) [36]. Based on each genBlastA hit, the software GeneWise v2.4.1 (RRID:SCR_015054) [37] was employed to predict the exact gene structure. Three programs for *de novo* gene prediction, Augustus v3.2.1 (RRID:SCR_008417) [38], GlimmerHMM v3.0.4 (RRID:SCR_002654) [39], and SNAP v2006-07-28 (RRID:SCR_002127) [40], were applied to explore coding regions in the assembly of *C. arbutifolia*. The software HISAT2 v2.0.1 (RRID:SCR_015530) [41] was used to map RNA-seq data to the chromosome-scaled *C. arbutifolia* assembly, and then, StringTie v1.2.2 (RRID:SCR_016323) [42] was used to assemble the transcripts. The program TransDecoder v3.0.1 (RRID:SCR_017647, https://github.com/TransDecoder/TransDecoder) was conducted to identify the candidate coding regions. Using the above approaches, all predicted gene models were integrated by EvidenceModeler (RRID:SCR_014659) [43] into a non-redundant set of gene structures that were finally refined with the Program to Assemble Spliced Alignments (PASA) v2.3.3 (RRID:SCR_014656) [44]. The protein-coding

genes were functionally annotated against two integrated SwissProt and TrEMBL databases using BLASTP (RRID:SCR_001010) [45] with E-value 1e-05. The software InterProScan v5.30 (RRID:SCR_005829) [46] was employed for protein domain annotation. For all genes, the GO terms were extracted using InterProScan v5.30 (RRID:SCR_005829) and the pathways were assigned against the KEGG database (release 84.0) using BLAST (RRID:SCR_004870).

Non-coding RNA prediction

Non-coding RNAs, including four types of transfer RNAs (tRNAs), ribosomal RNAs (rRNAs), small nuclear RNAs (snRNAs), and micro-RNAs (miRNAs), were predicted. tRNAs and rRNAs were discovered using tRNAscan-SE v1.3.1 (RRID:SCR_010835) [47] and BLASTn v2.2.24 (RRID:SCR_001598, E-value 1e-5) via the alignment to template rRNA and tRNA sequences of *Oryza* and *Arabidopsis*, respectively. SnRNAs and miRNAs were screened from the Rfam database (RRID: SCR_007891, release 12.0, http://eggnogdb.embl.de/) using INFERNAL v1.1.1 (RRID:SCR_011809, https://github.com/ebi-pf-team/interproscan).

Gene family analysis

The OrthoMCL v2.0.9 (RRID:SCR_007839) [48] clustering program was run on the proteomes of *C. arbutifolia*, *S. purpurea*, *S. suchowensis*, *S. viminalis*, *S. brachista*, *P. euphratica*, *P. tremuloides*, *P. tremula*, *P. pruinosa*, *P. trichocarpa*, *P. alba*, and *R. communis*. A phylogenetic tree for these 12 species was constructed using the identified single-copy gene families. From each family, four-fold degenerate sites were segregated and concatenated into one supergene. The phylogenetic tree was reestablished using the program MrBayes v3.1.2 (RRID:SCR_012067, https://github.com/NBISweden/MrBayes) with the model of GTR + gamma substitution. The program MCMCtree v4.4 in the PAML package (RRID:SCR_014932) [49] was used to estimate the divergence times among the 12 species, with the JC69 nucleotide substitution model and an independent rates clock. The calibration divergence

times between *S. purpurea* and *P. trichocarpa* (~35.6 MYA), and *R. communis* and *P. trichocarpa* (~80 MYA) were obtained from the TimeTree database (RRID: SCR_021162) [50]. Changes in gene family size within the phylogenetic tree were analyzed using CAFE v2.1 (RRID:SCR_005983) [51]. Positive selection genes in the *C. arbutifolia* genome were detected using the branch-site model incorporated in the PAML package (RRID:SCR_014932) [49] and a maximum likelihood ratio test based on the single copy genes. *C. arbutifolia* and the other 11 species (except *R. communis*) were determined as foreground and background branches of the phylogenetic tree, respectively. GO enrichment was derived using Fisher's exact test followed by Benjamini-Hochberg adjustments, with the cutoff of $P < 0.05$. WGD events were inferred based on the distribution of distance-transversion rate at 4DTv of paralogous gene pairs. The 4DTv transversion rates between all species pairs were calculated using an in-house Perl script.

Results and Discussion

Genome assembly

In total, 34.22 Gb with a ~101× HiFi read coverage were generated through whole-genome sequencing of *C. arbutifolia* using the PacBio Sequel platform (Supplementary Table S1). The PacBio reads were assembled and polished with ~111× Illumina paired-end reads (37.52 Gb, Supplementary Table S2), resulting in ~1.68 Mb of contig N50 (Table 1). Subsequently, another 27.81 Gb Dovetail Hi-C data with a ~82× depth were utilized to refine the genome assembly (Supplementary Table S2). Thus, a *C. arbutifolia* genome with a total size of 338.93 Mb was acquired and assigned to 19 pseudochromosomes (Fig. 1a), which is similar to those of *S. dunnii* [20] (328 Mb), *S. purpurea* (329.29 Mb, Table 1) and *S. brachista* [16] (339.58 Mb), but slightly smaller than those of *suchowensis* [17] (356.5Mb) and *S. viminalis* [18] (357.06 Mb). Compared with the genome sizes of *Populus* species, such as *P. trichocarpa* [8] (434.13 Mb), *P. euphratica* [9] (496.5Mb), *P. pruinosa* [10] (479.3 Mb), *P. tremula* [11] (390 Mb), *P. tremuloides* [11] (378 Mb), *P. alba* [12] (415.99 Mb), *P. alba* var. *pyramidalis* [13] (464 Mb), and *P. ilicifolia* [15]

(402 Mb), those of *C. arbutifolia* and *Salix* species are generally substantially smaller, which is consistent with early reports [17, 52]. The super-scaffolds number, super-scaffold N50, and maximum super-scaffold length were 304, ~16.46 Mb, and 31.95 Mb, respectively (Table 1). To evaluate assembly quality of the *C. arbutifolia* genome, 1390 core genes were identified in the OrthoDB embryophyta database, accounting for 96.6% of the total 1440 core genes, among which single-copy and duplicated genes represented 85.1% and 11.5%, respectively (Supplementary Table S3). The features of assembled genomes of different Salicaceae species are illustrated in Table 1.

Repetitive sequence identification

Among the assembled genome sequences of *C. arbutifolia*, a total of ~143.47 Mb (42.34%) repeat element sequences were screened, of which tandem and interspersed repeats accounted for 8.47% and 38.21%, respectively (Supplementary Table S4). Among the interspersed repeats, three types of repetitive elements, including Class I (retrotransposons), Class II (DNA transposons), and unclassified elements, representing 38.21% of the genome assembly, were identified (Supplementary Table S5). The long terminal repeat (LTR) retrotransposons represented the most frequent among Class I repetitive sequences, with Gypsy and Copia LTR retrotransposons accounting for 14.90% and 14.25%, respectively, whereas long and short interspersed nuclear elements represented approximately 3% of the genome size. The insertion time of LTR retrotransposons was predicted by detecting the sequence divergence at both ends of impact LTRs. As shown in Fig. 1b, a surge of retrotransposon amplification was detected in *C. arbutifolia* approximately 0.472 million years ago (MYA), indicating an expansion event in the recent period of genome evolution.

Gene annotation

Through a combined prediction strategy of *ab initio*, homologous protein, and transcriptome, 33,229 protein-coding genes were predicted (Supplementary Table S6). Of these, 31,618 (95.15%) were successfully annotated in diverse

databases, including NCBI nr, Swissprot, KEGG, TrEMBL, and InterPro, whereas the remaining 1611 (4.85%) genes had no significant correspondence with sequences in public databases (Supplementary Table S6, Fig. 1c). The reason for the overall smaller genomes of the *Salix* species has been suggested to be the faster evolution speed of willows, which reduces the predicted gene number [17, 52]. However, we found that there is no linear correspondence between the genome size and number of predicted genes in the Salicaceae species. For example, 36,490 genes have been identified in *S. viminalis* [18] in an assembled genome of 357.06 Mb. The *S. viminalis* genome is smaller in size but harbors a larger number of genes than those of *P. pruinosa* [10] (35,131 genes), *P. alba* [12] (32,963 genes) and *P. ilicifolia* [15] (33,684 genes). *S. brachista* [16] has a slightly larger genome (339.58 Mb), but smaller number of predicted genes (30,209) than *C. arbutifolia* (338.93 Mb; 33,229 genes) and *S. purpurea* (329.29 Mb; 35,125 genes). Without a doubt, the efficiency of genome assembly and the strategy used for gene mining are essential factors affecting the numbers of predicted genes in different species. The mean length of the predicted protein-coding genes was 3156 bp, with 5.02 exons per gene, and the average lengths of exons and introns were 233 bp and 446 bp, respectively (Supplementary Table S7). Non-coding RNAs in the *C. arbutifolia* genome were explored and annotated, and comprised 239 miRNAs, 697 tRNAs 10,043 rRNAs, and 495 snRNAs (Supplementary Table S8).

Phylogenetic relationship analysis

The protein-coding genes of 11 Malpighiales species, including *S. purpurea*, *S.suchowensis*, *S. viminalis*, *S. brachista*, *P. trichocarpa*, *P. tremuloides*, *P. tremula*, *P. pruinosa*, *P. euphratica*, *P. alba*, and *Ricinus communis*, were collected from relevant databases and clustered into 30,618 gene families together with the protein-coding genes of *C. arbutifolia* (Supplementary Table S9, Fig. 2a). The analysis of gene family intersection exhibited that 11,308 gene families were shared by the 11 Salicaceae species, but not *R. communis* (Fig. 2b). For *C. arbutifolia*, 28,512 genes were assigned to 18,729 genes families, of which 184 families, containing 1,750 genes in total, were specific when compared with the 11 other Malpighiales species (Supplementary Table S9). These genes were significantly enriched

in the Gene Ontology (GO) terms “DNA binding”, “ribonucleoside binding”, and “DNA-directed 5'-3' RNA polymerase activity” with FDR < 0.05 (Supplementary Table S10).

A phylogenetic tree was constructed for the 12 Malpighiales species, considering *R. communis* as an outgroup (Fig. 2c). The divergence time between *Chosenia* and *Populus* was assessed to be around 15.3 MYA, and *C. arbutifolia* was separated from the four *Salix* species around ~6.6 MYA, indicating that *C. arbutifolia* was the first species to differentiate from *Populus* and may be a transitional species between poplars and willows. It preserved some poplar characteristics, such as the haploid number (n = 19, most of the tree species in *Salix* are polyploid), wind pollination, and absence of glands. In addition, in previous reports on *S. brachista* [16] and *S. dunnii* [20] demonstrate exactly the same relationships and similar divergence times among the above-mentioned species, indicating that *S. suchowensis* may have evolved substantially further than other *Salix* species owing to a stronger purifying selection [17, 52]. However, the family Salicaceae comprises more than 600 species worldwide, and limited available genome data of Salicaceae species were analyzed in this study. Thus, to completely clarify the phylogenetic history of this family, more species should be added in future.

Compared with the most recent common ancestor (MRCA) of *Chosenia* and *Salix*, *C. arbutifolia* showed 72 and 85 expansion and contraction events of each gene family, respectively (Fig. 2c). The results of GO enrichment analysis revealed that among the expanded genes, 29 genes were associated with “heme binding” and “oxidation-reduction process”, and 22 genes were involved in “iron ion binding” (Supplementary Table S11). Among the contracted genes, 93 genes were related to “ATP binding”, and 88 genes were responsible for “protein kinase activity” and “protein phosphorylation” (Supplementary Table S11). Positive selection genes (PSGs) were detected using single-copy gene sets of the 12 species. In *C. arbutifolia*, a total of 89 PSGs were detected, of which six and five PSGs were enriched in the GO terms of “integral component of membrane” and “catalytic activity”, respectively, whereas three PSGs were related to both “ATP binding” and “nucleic acid binding” (Supplementary Table S12).

Whole-genome duplication analysis

Based on the fourfold degenerate sites (4DTv) approach, whole-genome duplication (WGD) events were deduced. After the speciation between *C. arbutifolia* and *Arabidopsis thaliana* (4DTv = 0.64), a common salicoid WGD event occurred (4DTv = 0.13). The divergence between *C. arbutifolia* and *P. trichocarpa* emerged at the peak of 4DTv ~ 0.05, followed by *C. arbutifolia* and *S. purpurea* (4DTv = 0.02), which is in consistent with the results of phylogenetic analysis (Fig. 2d). After the differentiation of the Salicaceae species, there was no obvious evidence of a *C. arbutifolia*-specific WGD.

Genome collinearity analysis

Genome collinearity among *C. arbutifolia*, *S. purpurea*, *S. suchowensis*, and *P. trichocarpa* was analyzed. The syntenic regions showed that most chromosomes were highly conserved among the Salicaceae species, except for a large interchromosomal-recombination between chromosomes one and sixteen (Fig. 3a). Furthermore, the whole chromosomes of *C. arbutifolia* and the two *Salix* species were highly collinear (Fig. 3b). Together, these results indicated that main chromosomal fissions and fusions have occurred during the evolution of Salicaceae, resulting in a genera divergence of Salicaceae. Like in other *Salix* species, such as *S. brachista* [16], *S. suchowensis* [17, 52], and *S. dunnii* [20], most of the chromosomes of *C. arbutifolia* were highly conserved with *P. trichocarpa*, except for chromosomes one and sixteen, where a large interchromosomal recombination was discovered (Fig. 3a). It has been reported that the chromosomal fusions and fissions that emerged in *Populus* after a lineage-specific salicoid duplication gave rise to the divergence of the two genera, *Populus* and *Salix* [53]. Nevertheless, recombination modes are quite different between *S. suchowensis* and other *Salix* species. Chromosome sixteen of *S. suchowensis* entirely originated from a partial chromosome one of *P. trichocarpa*, and chromosome 1 of *S. suchowensis* was comprised of the remaining part of chromosome one and the entire chromosome sixteen of *P. trichocarpa* [17]. However, in our study, chromosome sixteen of *C. arbutifolia* was fused with a partial chromosome one and the entire

chromosome sixteen of *P. trichocarpa*, and chromosome one of *C. arbutifolia* comprised the remaining part of *P. trichocarpa* chromosome one. This difference was confirmed by collinearity analysis (Fig. 3b) and the same phenomenon was also detected in *S. brachista* [16] and *S. dunnii* [20].

Conclusions

Although multiple genomes of *Populus* and *Salix* species have been reported, we sequenced and assembled a genome of the taxonomically difficult species *C. arbutifolia* that belongs to the monotypic genus *Chosenia* for the first time by using PacBio HiFi reads, Hi-C chromatin contact maps, and Illumina short reads. As a significant supplementary for the family Salicaceae, the assembled genome took a deep insight into the genomic architecture of *C. arbutifolia* and revealed the systematic evolution and phylogenetic relationships with other Salicaceae species. Given the limited genomic resources in the public databases, it is worthwhile to take full advantage of more available genomic information for further studies. Overall, our results lay a solid foundation for genetic and genomic research on Salicaceae species in future.

Data Availability

The genome assembly and all the sequencing data have been deposited in GenBank database under the accession number PRJNA788330.

Additional Files

Supplementary Table S1. Statistics of Pacbio HiFi data

Supplementary Table S2. Statistics of Illumina data

Supplementary Table S3. Statistics of the *C. arbutifolia* assembly gene-space with the 1440 BUSCO embryophyta gene set

Supplementary Table S4. General statistics of the repeats in *C. arbutifolia* genome

Supplementary Table S5. Interspersed repeats (TEs) content in the assembled *C. arbutifolia* genome

Supplementary Table S6. Functional annotation of the predicted genes for *C. arbutifolia*

Supplementary Table S7. Statistics of the predicted protein-coding genes in different species

Supplementary Table S8. Non-coding RNAs in the *C. arbutifolia* genome

Supplementary Table S9. Statistics of gene families of the twevle Malpighiales species

Supplementary Table S10. GO enrichment of the specific genes in *C. arbutifolia*

Supplementary Table S11. GO enrichment of expanded and contracted genes in *C. arbutifolia*

Supplementary Table S12. GO enrichment of positive selection genes in *C. arbutifolia*

Abbreviations

4DTv: 4-fold degenerate sites; BLAST: Basic Local Alignment Search Tool; BUSCO: Benchmarking Universal Single-Copy Orthologs; GO: Gene Ontology; HiFi: High-Fidelity; KEGG: Kyoto Encyclopedia of Genes and Genomes; LTR: long terminal repeat; miRNAs: micro-RNAs; MRCA: most recent common ancestor; MYA: million years ago; PSGs: positive selection genes; RLKs: receptor-like kinases; rRNAs: ribosomal RNAs; RNA-seq: RNA sequencing; PASA: Program to Assemble Spliced Alignments; SMRT: Single-Molecule Real-time Sequencing; snRNAs: small nuclear RNAs; tRNAs: transfer RNAs; WGD: whole-genome duplication.

Funding

This work was financially supported by the National Natural Science Foundation of China (Grant No. 31670662) and Independent Scientific Research Project of Jiangsu Academy of Forestry (Grant No. ZZKY202101).

Competing Interests

The authors declare that they have no conflict of interest.

Authors' Contribution

XDH and QZ conceived and designed the experiments. XDH wrote and revised the manuscript. YW, JML, JWZ, JZ, JL, ZYJ and YCN analyzed the data. BSW and WWW collected the samples. JZ processed the data. All authors have read and approved the final manuscript.

Acknowledgements

The authors are grateful to Jun Ren in Jilin Academy of Forestry for their assistance with sample collection. Special thanks are due to Prof. Tongming Yin, Siming Gan and anonymous reviewers for their valuable comments on the manuscript.

References

1. Wang, Z, Fang, CF. Salicaceae. In Flora Republicae Popularis Sinicae. Science Press; 1984. p. 79-81.

2. Kadis, I. Chosenia: an amazing tree of Northeast Asia. *Arnoldia* 2005;**63**(3):8-17.

3. Nakai, T. *Chosenia*, a new genus of Salicaceae. *Bot Mag* 1920;**34**:66-9.

4. Moskalyuk, TA. *Chosenia arbutifolia* (Salicaceae): life strategies and introduction perspectives. *Siberian J For Sci* 2016;**3**:34-45.

5. He, XD, Wang, Y, Zheng, JW, *et al*. Phylogenetic analysis of *Chosenia arbutifolia* (Pall.) A. Skv. in Salicaceae using complete chloroplast genome sequence. *Ann For Res* 2022;**65**(1): 3-16.

6. Tu, ZY. Breeding and cultivation of *Salix*. Jiangsu Science and Technology Press; 1982. p. 154-196.

7. Hanley, S, Mallott, M, Karp, A. Alignment of a *Salix* linkage map to the *Populus* genomic sequence reveals macrosynteny between willow and poplar genomes. *Tree Genet Genomes* 2006;**3**(1): 35-48.

8. Tuskan, GA, DiFazio, S, Jansson, S, *et al*. The genome of black cottonwood, *Populus trichocarpa* (Torr. & Gray). *Science* 2003;**313**(5793):1596-1604.

9. Ma, T, Wang, JY, Zhou, GK, *et al*. Genomic insights into salt adaptation in a desert poplar. *Nat Commun* 2013;**4**(1):2797.

10. Yang, WL, Wang, K, Zhang, J, *et al*. The draft genome sequence of a desert tree *Populus pruinosa*. *GigaScience* 2017;**6**(9):1-7.

11. Lin, YC, Wang, J, Delhomme, N, *et al*. Functional and evolutionary genomic inferences in *Populus* through genome and population sequencing of American and European aspen. *PNAS* 2018;**115**(46):E10970-8.

12. Liu, YJ, Wang, XR, Zeng, QY. *De novo* assembly of white poplar genome and genetic diversity of white poplar population in Irtysh River basin in China. *Sci China Life Sci* 2019;**62**(5): 609-18.

13. Ma, JC, Wan, DS, Duan, BB, *et al*. Genome sequence and genetic transformation of a widely distributed and cultivated poplar. *Plant Biotechnol J* 2019;**17**(2):451-60.

14. Qiu, DY, Bai, SL, Ma, JC, *et al*. The genome of *Populus alba* × *Populus tremula* var. *glandulosa* clone 84K. *DNA Res* 2019;**26**(5):423-31.

15. Chen, ZY, Ai, FD, Zhang, JL, *et al*. Survival in the Tropics despite isolation, inbreeding and asexual reproduction: insights from the genome of the world's southernmost poplar (*Populus ilicifolia*). *Plant J* 2020;**103**(1):430-42.

16. Chen, JH, Huang, Y, Brachi, B, *et al*. Genome-wide analysis of cushion willow provides insights into alpine plant divergence in a biodiversity hotspot. *Nat Commun* 2019;**10**(1): 5230.

17. Wei, SY, Yang, YH, Yin, TM. The chromosome-scale assembly of the willow genome provides insight into Salicaceae genome evolution. *Horticul Res* 2020;**7**:45.

18. Almeida, P, Proux-Wera, E, Churcher, A, *et al*. Genome assembly of the basket willow, *Salix viminalis*, reveals earliest stages of sex chromosome expansion. *BMC Biol* 2020;**18**: 78.

19. Zhang, J, Yuan, HW, Li, YJ, *et al*. Genome sequencing and phylogenetic analysis of allotetraploid *Salix matsudana* Koidz. *Horticul Res* 2020;**7**:201.

20. He, L, Jia, KH, Zhang, RG, *et al*. Chromosome-scale assembly of the genome of *Salix dunnii* reveals a male-heterogametic sex determination system on chromosome 7. *Mol Ecol Resour* 2021;**21**(6):1966-82.

21. Chen, JH, Hao, ZD, Guang, XM, *et al*. Liriodendron genome sheds light on angiosperm phylogeny and species-pair differentiation. *Nat Plants* 2019;**5**:18-25.

22. Ma, QY, Sun, TL, Li, SS, *et al*. The *Acer truncatum* genome provides insights into nervonic acid biosynthesis. *Plant J* 2020;**104**(3):662-78.

23. Chen, S, Wang, YC, Yu, LL, *et al*. Genome sequence and evolution of *Betula platyphylla*. *Hortic Res* 2021;**8**:37.

24. Cao, YB, Sun, GL, Zhai, XQ, *et al*. Genomic insights into the fast growth of paulownias and the formation of *Paulownia* witches' broom. *Mol Plant* 2021;**14**(10):1668-82.

25. Xiong, XY, Gou, JB, Liao, QG, *et al*. The *Taxus* genome provides insights into paclitaxel biosynthesis. *Nat Plants* 2021;**7**:1026-36.

26. He, XD, Wang, Y, Zheng, JW, *et al*. Full-length transcriptome characterization and comparative analysis of *Chosenia arbutifolia*. *Forests* 2022;**13**(4):543.

27. Garg, S, Fungtammasan, A, Carroll, A, *et al*. Chromosome-scale, haplotype-resolved assembly of human genomes. *Nat Biotechnol* 2021;**39**(3):309-12.

28. Walker, BJ, Abeel, T, Shea, T, *et al*. Pilon: an integrated tool for comprehensive microbial variant detection and genome assembly improvement. *PLoS ONE* 2014;**9**(11):e112963.

29. Servant, N, Varoquaux, N, Lajoie, BR, *et al*. HiC-Pro: an optimized and flexible pipeline for Hi-C data processing. *Genome Biol* 2015;**16**:259.

30. Durand, NC, Shamim, MS, Machol, I, *et al*. Juicer provides a one-click system for analyzing loop-resolution Hi-C experiments. *Cell Syst* 2016;**3**(1):95-8.

31. Dudchenko, O, Batra, SS, Omer, AD, *et al*. *De novo* assembly of the *Aedes aegypti* genome using Hi-C yields chromosome-length scaffolds. *Science* 2017;**356**(6333):92-5.

32. Simão, FA, Waterhouse, RM, Ioannidis, P, *et al*. BUSCO: assessing genome assembly and annotation

completeness with single-copy orthologs. *Bioinformatics* 2015;**31**(19): 3210-12.

33. Benson, G. Tandem repeats finder: a program to analyze DNA sequences. *Nucleic Acids Res* 1999;**27**(2):573-80.

34. Xu, Z, Wang, H. LTR_FINDER: an efficient tool for the prediction of full-length LTR retrotransposons. *Nucleic Acids Res* 2007;**35**(suppl_2):W265-8.

35. Bao, WD, Kojima, KK, Kohany, O. Repbase Update, a database of repetitive elements in eukaryotic genomes. *Mobile DNA* 2015;**6**:11.

36. She, R, Chu, JS, Wang, K, *et al*. genBlastA: enabling BLAST to identify homologous gene sequences. *Genome Res* 2008;**19**(1):143-9.

37. Birney, E, Clamp, M, Durbin, R. GeneWise and Genomewise. *Genome Res* 2004;**14**(5):988-95.

38. Stanke, M, Keller, O, Gunduz, I, *et al*. AUGUSTUS: *ab initio* prediction of alternative transcripts. *Nucleic Acids Res* 2006;**34**(suppl_2):W435-9.

39. Majoros, WH, Pertea, M, Salzberg, SL. TigrScan and GlimmerHMM: two open source *ab initio* eukaryotic gene-finders. *Bioinformatics* 2004;**20**(16):2878-9.

40. Korf, I. Gene finding in novel genomes. *BMC Bioinformatics* 2004; **5**:59.

41. Kim, D, Langmead, B, Salzberg, SL. HISAT: a fast spliced aligner with low memory requirements. *Nat Methods* 2015;**12**(4):357-60.

42. Kovaka, S, Zimin, AV, Pertea, GM, *et al*. Transcriptome assembly from long-read RNA-seq alignments with StringTie2. *Genome Biol* 2019;**20**(1):278.

43. Haas, BJ, Salzberg, SL, Zhu, W, *et al.* Automated eukaryotic gene structure annotation using EVidenceModeler and the program to assemble spliced alignments. *Genome Biol* 2008;**9**:R7.

44. Haas, BJ, Delcher, AL, Mount, SM, *et al*. Improving the Arabidopsis genome annotation using maximal transcript alignment assemblies. *Nucleic Acids Res* 2003;**31**(19):5654-66.

45. McGinnis, S, Madden, TL. BLAST: at the core of a powerful and diverse set of sequence analysis tools. *Nucleic Acids Res* 2004;**32**(suppl_2):W20-5.

46. Quevillon, E, Silventoinen, V, Pillai, S, *et al*. InterProScan: protein domains identifier. *Nucleic Acids Res* 2005;**33**(suppl_2):W116-20.

47. Lowe, TM, Eddy, SR. tRNAscan-SE: a program for improved detection of transfer RNA genes in genomic sequence. *Nucleic Acids Res* 1997;**25**(5):955-64.

48. Li, L, Stoeckert, CJ, Roos, DS. OrthoMCL: identification of ortholog groups for eukaryotic genomes. *Genome Res* 2003;**13**(9):2178-89.

49. Yang, ZH. PAML: a program package for phylogenetic analysis by maximum likelihood. *Bioinformatics* 1997;**13**(5):555-6.

50. Hedges, SB, Dudley, J, Kumar, S. TimeTree: a public knowledge-base of divergence times among organisms. *Bioinformatics* 2006;**22**(23):2971-2.

51. Bie, TD, Cristianini, N, Demuth, JP, *et al*. CAFE: a computational tool for the study of gene family evolution. *Bioinformatics* 2006;**22**(10):1269-71.

52. Hou, J, Wei, SY, Pan, HX, *et al*. Uneven selection pressure accelerating divergence of *Populus* and *Salix*. *Horticul Res* 2019;**6**:37.

53. Hou, J, Ye, N, Dong, ZY, *et al*. Major chromosomal rearrangements distinguish willow and poplar after the ancestral “Salicoid” genome duplication. *Genome Biol Evol* 2016;**8**(6): 1868-75.

Table 1: Comparison of Salicaceae assemblies

Assembly feature	*C. arbutifolia*	*S. suchowensis*	*S. purpurea*	*P. trichocarpa*
Size (Mb)	338.93	356.5	329.29	434.13
No. of super-scaffolds	304	1,201	348	1,446
Contig N50 (bp)	1,682,645	263,908	5,083,238	552,806
Super-scaffold N50	16,460,042	16,776,717	14,688,223	19,465,461
Longest super-scaffold (Mb)	31.95	34.98	32.43	50.50
No. of protein coding genes	33,229	36,937	35,125	41,335
Complete BUSCOs (%)	96.6	94.8	96.2	97.4

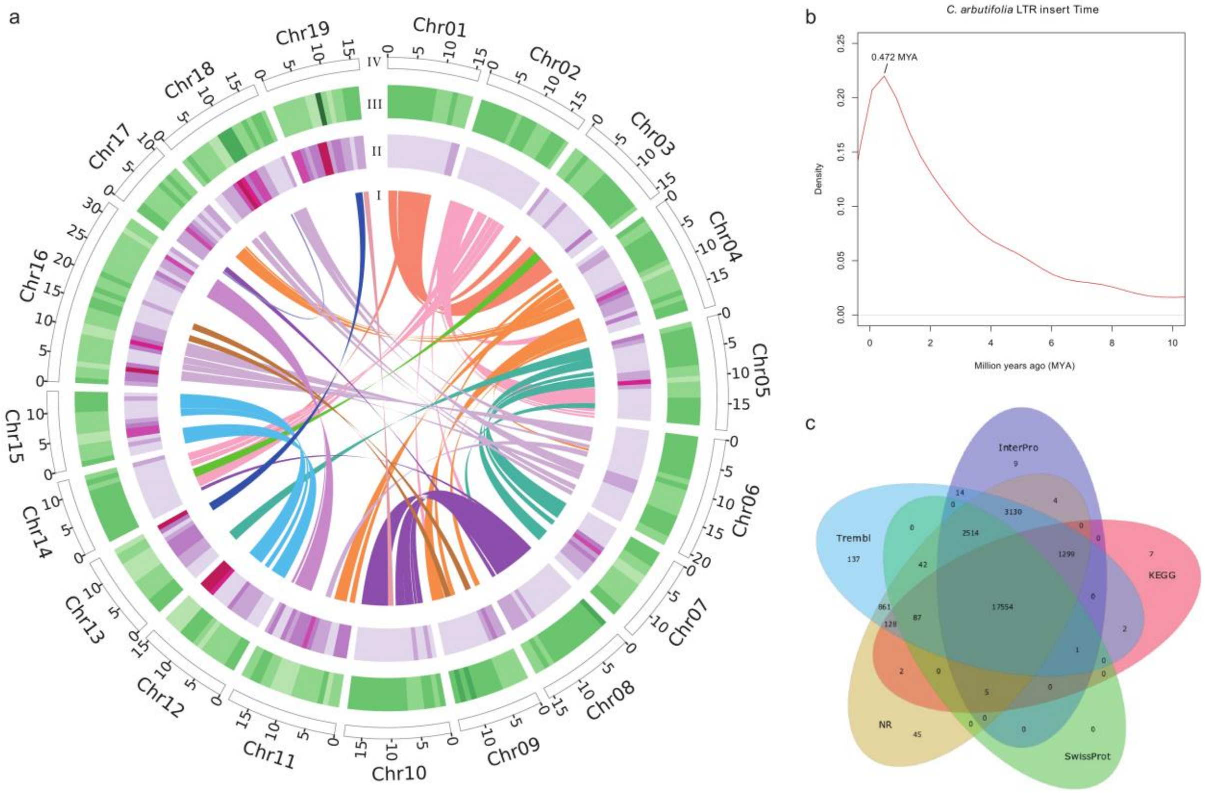

Figure 1: *C. arbutifolia* genome characeristics. **a,** Genome circos plot. I: Collinear regions within the *C. arbutifolia* assembly; II: Percentage of transposable elements in 1 Mb sliding windows; III: Gene density in 1Mb sliding windows; IV: Chromosomes length in Mb. **b,** Insertion times of LTR retrotransposons. **c,** Venn diagram showing genes shared among different annotated datasets.

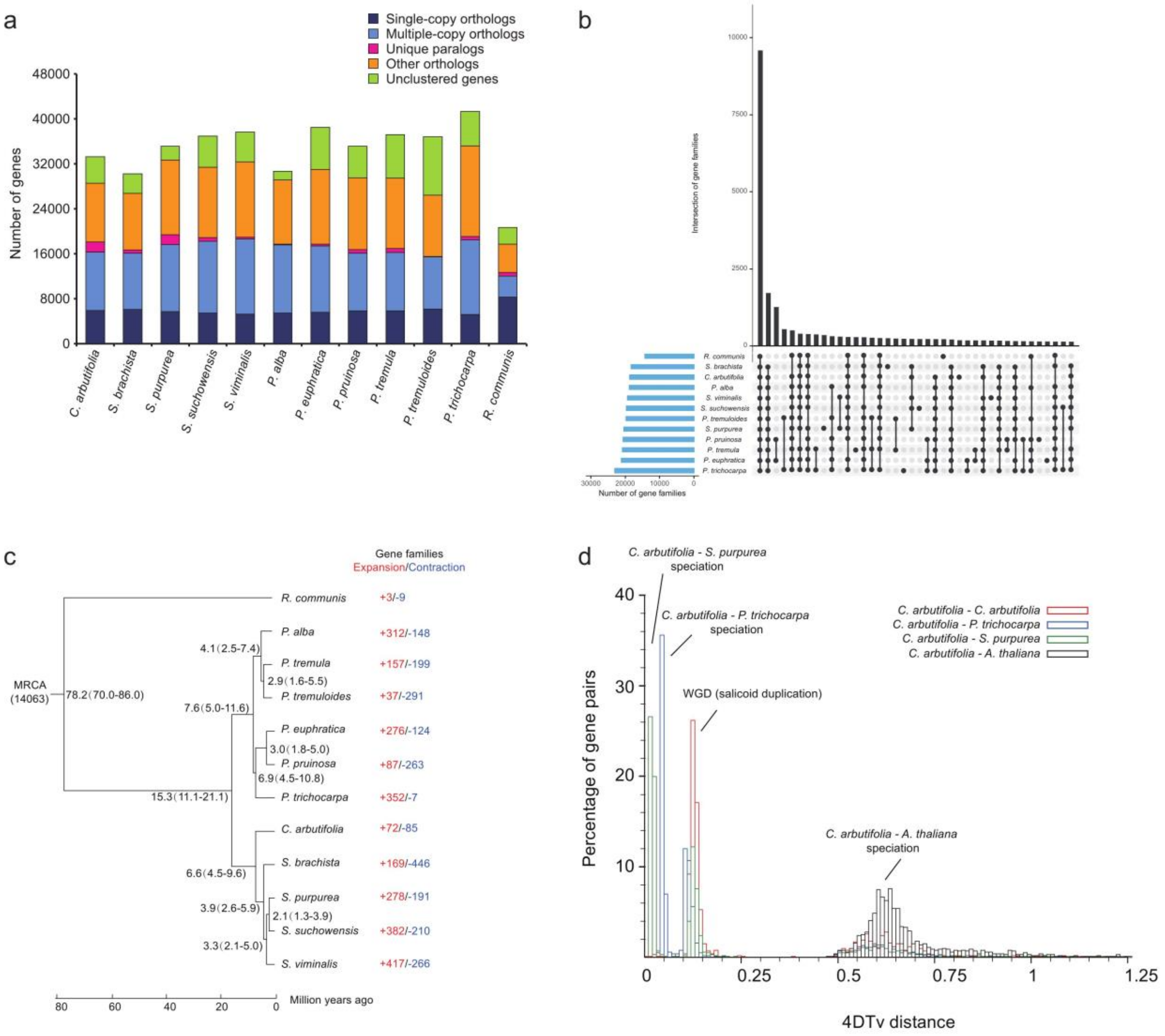

Figure 2: Genome comparison of different Malpighiales species. **a,** Protein orthology comparison in genomes of the indicated 12 species. **b,** Intersections of gene families among the 12 species. Rows and columns represent gene families and intersections, respectively. Black and gray circles indicate the existence or absence of a given intersection. Vertical black lines connecting black circles in each column represent the column based relationship. The bar chart located at the top of the matrix indicate the intersection size. The horizontal bar chart on the left side of the matrix indicates the size of gene family. **c,** Phylogenetic tree of the 12 species. Numbers (black) on nodes indicate the differentiation time, and error ranges are shown in parentheses. **d,** Genome duplication in the *C. arbutifolia* genome revealed by 4DTv analysis.

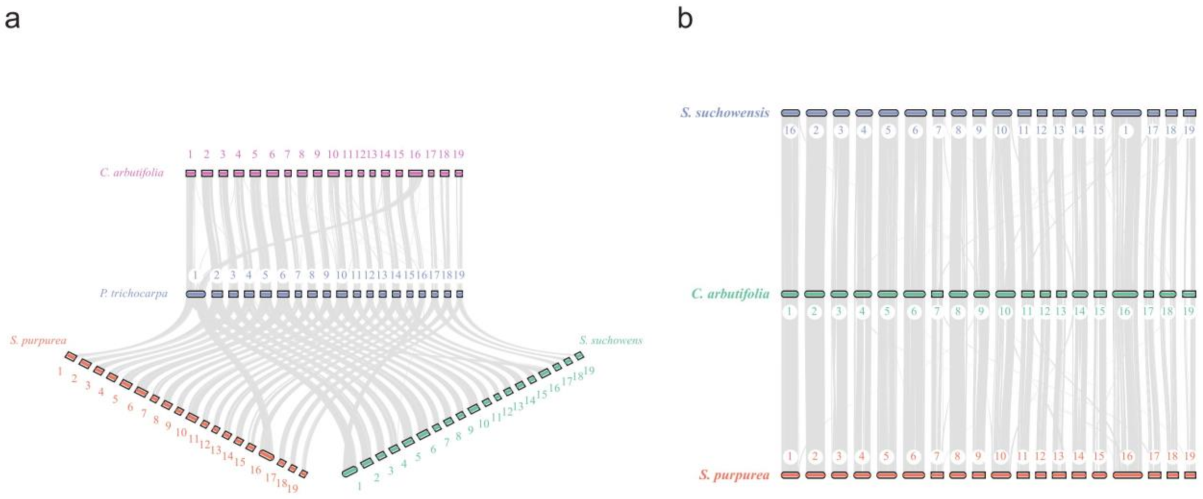

Figure 3: Synteny analysis. **a,** Synteny analysis of *C. arbutifolia*, *S. purpurea*, *S. suchowensis*, and *P. trichocarpa*. **b,** Synteny analysis of *C. arbutifolia*, *S. purpurea*, and *S. suchowensis*. Macrosynteny connecting blocks of >30 one-to-one gene pairs are shown.

Additional file

Click here to access/download
Supplementary Material
Supplementary tables.xlsx
