# Supplementary material for: The whole-genome assembly of an endangered Salicaceae species: Chosenia arbutifolia (Pall.) A. Skv
Source: Gigascience. 2022 Nov 14;11:giac109. doi: 10.1093/gigascience/giac109 (PMC9661892; doi:10.1093/gigascience/giac109)
Supplement: giac109_GIGA-D-22-00145_Revision_1 [file giac109_giga-d-22-00145_revision_1.pdf]

GigaScience

The whole-genome assembly of an endangered Salicaceae species: Chosenia arbutifolia (Pall.) A. Skv.

--Manuscript Draft--

**Manuscript Number:**	GIGA-D-22-00145R1
**Full Title:**	The whole-genome assembly of an endangered Salicaceae species: Chosenia arbutifolia (Pall.) A. Skv.
**Article Type:**	Data Note
**Funding Information:**	National Natural Science Foundation of China (31670662) — Dr. Xudong He<br>Independent Scientific Research Project of Jiangsu Academy of Forestry (ZZKY202101) — Prof. Baosong Wang
**Abstract:**	Background<br><br>As a fast-growing tree species, Chosenia arbutifolia has a unique, but controversial taxonomic status in the family Salicaceae. Despite its importance as an industrial material, in ecological protection, and in landscaping, C. arbutifolia is seriously endangered in Northeast China because of artificial destruction and its low reproductive capability.<br><br>Results<br><br>To clarify its phylogenetic relationships with other Salicaceae species, we assembled a high-quality chromosome-level genome of C. arbutifoli a using PacBio HiFi reads and Hi-C sequencing data, with a total size of 338.93 Mb and contig N50 of 1.68 Mb. Repetitive sequences, which accounted for 42.34% of the assembly length, were identified. In total, 33,229 protein-coding genes and 11,474 small ncRNAs were predicted. Phylogenetic analysis suggested that C. arbutifolia and poplars diverged approximately 15.3 million years ago, and a large interchromosomal recombination between C. arbutifolia and other Salicaceae species was discovered.<br><br>Conclusions<br><br>Our study provides insights into the genome architecture and systematic evolution of C. arbutifolia , as well as comprehensive information for germplasm protection and future functional genomic studies.
**Corresponding Author:**	Xudong He<br>Jiangsu Academy of Forestry<br>Nanjing, Jiangsu CHINA
**Corresponding Author Secondary Information:**	
**Corresponding Author's Institution:**	Jiangsu Academy of Forestry
**Corresponding Author's Secondary Institution:**	
**First Author:**	Xudong He
**First Author Secondary Information:**	
**Order of Authors:**	Xudong He<br>Yu Wang<br>Jinmin Lian<br>Jiwei Zheng<br>Jie Zhou<br>Jiang Li

Powered by Editorial Manager® and ProduXion Manager® from Aries Systems Corporation

	Zhongyi Jiao
	Yongchao Niu
	Weiwei Wang
	Jun Zhang
	Baosong Wang
	Qiang Zhuge
**Order of Authors Secondary Information:**	
**Response to Reviewers:**	Dear Editor,<br><br>We sincerely thank you and the reviewers for your valuable comments and instructive advice, which help us to improve and revise our manuscript. We have made detailed changes accordingly. Our point-by-point responses to the reviewers' comments are as follows for your consideration.<br><br>Response to Reviewer #1:<br><br>He et al. reported the genome assembly and characterization of an endangered Salicaceae species, Chosenia arbutifolia, with significant values in phylogenetic and evolutionary study of the important family. The methods are appropriate, the genome data are generally in high quality, and genome characterization revealed the key findings in Chosenia phylogeny and genome history.<br><br>Recommend to accept with minor revision.<br><br>Minor Comments:<br>page 4, line 23, why S. suchowensis was collected? Is it a part of this study reported here?<br>Answer 1: Sorry for the misleading. In the first place there were some biological analyses in the manuscript. We have modified this part, please see page 5, line 2.<br><br>page 5, line 2, "transported back to the laboratory", please be specific, as we have many many labs.<br>Answer 2: Yes, the institute name was added, please see page 5, line 4.<br><br>page 6, line 2-4, please provide full information on genome assembly, the parameters, settings, main considerations.<br>Answer 3: Yes, the DipAsm software depends on packages of Peregrine, 3d-dna, minimap2, DeepVariant, whatshap and hapcut2. The detailed parameters and settings used for DipAsm assembly were described in Supplementary Table 1. Please see page 6, line 5.<br><br>page 6, line 13, I do not think BUSCO could provide assessment on sequence integrity. It could be a kind of assessment on completeness of the genome assembly.<br>Answer 4: Yes, the word "integrity" was change to "completeness". Please see page 6, line 16.<br><br>page 7, line 18, where are the RNA-seq data from? Please provide full information.<br>Answer 5: The RNA was extracted from the leaves of C. arbutifolia using An Omega Plant RNA Kit (Omega Bio-tek, Norcross, GA, USA). Please see page 5, line 6, the section of "Plant materials and nucleic acid extraction".<br><br>page 9, line 16-17, the contig N50 is about 1.68 Mb which is not good as expected. Given the small genome size, the high sequencing depth of HiFi reads, it is necessary to check what happens there, why you just got such a low N50? Could you provide length distribution of your HiFi data? Or any other clues you have?<br>Answer 6: Yes, the length distribution of HiFi data was shown in figure 1a. The Kmer analysis conducted by GCE (Binghang Liu, et. al. 2013) showed that C. arbutifolia is a highly heterozygous (2.5% heterozygous rate) and highly repetitive (49.17% repeat content) species, which would affect the quality of the assembly, resulting a relatively low N50 (figure 1c).

Powered by Editorial Manager® and ProduXion Manager® from Aries Systems Corporation

Reference:
Binghang Liu, Yujian Shi, Jianying Yuan, et al. Estimation of genomic characteristics by analyzing k-mer frequency in de novo genome project. arXiv.org arXiv: 1308.2012. (2013)

page 10, line 3-4, please provide bootstrap values for the tree you presented here.
Answer 7: Yes, the bootstrap values were added, please see Figure 3c.

page 14, line 9, took -> provides or shed?
Answer 8: Yes, the word “took” was changed to “shed”, please see page 15, line 5.

Response to Reviewer #2:

The article is written in the correct language, the methods are applied in accordance with the standards used in plant genomics. On the other hand, it is a pity that the authors did not include Salix dunnii in their phylogenetic studies, even though the genome has been available since December 2020.

Some minor statements need correction:

Page 3 - line 6, statements need clarification - willows (Salix) are also wind pollinated, in particular some apline species exhibit both wind and insect pollination https://doi.org/10.1139/b99-003
Answer 1: Yes, this statement was adjusted, please see page 3, line 6.

Page 14 - line 17, the BioProject number is not found in the NCBI database, is it correct?
Answer 2: The BioProject number was correct, and you can access the data by the link: https://www.ncbi.nlm.nih.gov/bioproject/PRJNA788330

Response to Reviewer #3:

The manuscript titled "The whole-genome assembly of an endangered Salicaceae species: Chosenia arbutifolia (Pall.) A. Skv." provides a chromosome-scale genome assembly and annotation for C. arbutifolia, a Salix genome of great economic and ecological importance. In addition, by comparing with other genomes, the authors characterized the evolutionary relations between C. arbutifolia and Salicaceae species. Overall, this article provided informative and useful genomic resources for a Chosenia species, but the authors should provide additional details in the use of their methods and descriptions for results which lack several critical details listed below.

Major concerns:
1. Page 7, line 11-12: This paper selected seven plant genomes for homologous gene prediction, but only Populus trichocarpa belongs to Salicaceae. Now that various Populus and Salix genomes have been published and some of them have been applied for gene family analysis, why didn't choose them for gene prediction? How do you evaluate the correctness of this part of result?
Answer 1: Thank you for the good question. We selected seven plant genomes for homologous gene prediction, among which two species (Salix purpurea and Populus trichocarpa) belongs to Salicaceae. As the genus Chosenia is a unique member of the family Salicaceae along with Populus and Salix, the genetic composition of C. arbutifolia may be different from that of other Salicaceae species, so we selected not only Salix and Populus species but also other closely related plants for homology annotation. In addition, the complete BUSCOs of the predicted proteins is about 97.3%, indicating the high quality of the annotated genes, please see Supplementary Table 8. We also added a statement in the text, please page 11, line 19.

2. Page 9, line 23: What is the genomic reason for the difference between C. arbutifolia and other Populus species now that significant size changes were discovered?
Answer 2: The genome size of C. arbutifolia and most diploid Salix species are substantially smaller than Populus species. A repeat-driven genome expansion event was discovered in P. euphratica in early report (Zhang et al. 2020), which may be a possible reasons for the difference in genome size between C. arbutifolia and other

Powered by Editorial Manager® and ProduXion Manager® from Aries Systems Corporation

Populus species. In addition, some previous studies have reported that Salix species evolved faster than Populus species and might be subjected to stronger purifying selection than poplars, resulting in a reduced number of predicted genes and an overall smaller genome size (Dai et al. 2014; Hou et al. 2019; Wei et al. 2020)

Reference:
Dai et al. The willow genome and divergent evolution from poplar after the common genome duplication. Cell Res. 2014, 24: 1274.
Hou et al. Uneven selection pressure accelerating divergence of Populus and Salix. Horticul Res. 2019, 6:37.
Wei et al. The chromosome-scale assembly of the willow genome provides insight into Salicaceae genome evolution. Horticul Res. 2020, 7:45.
Zhang et al. Improved genome assembly provides new insights into genome evolution in a desert poplar (Populus euphratica). Mol Ecol Resour. 2020, 20(3): 781-794.

3. Page 10, line 3: This article applied BUSCO for genome assembly evaluation, but obviously this is not enough. How is this genome supported by the sequencing data, such as the mapping rate/data coverage of the NGS/hifi reads? What is the error rate, i.e. the homozygous snp ratio? Besides, what is the alignment result of RNA-seq data? Are there any assembled contigs that may belong to potential bacterial or virual pollution?
Answer 3: Thanks for your valuable suggestions. The NGS reads' mapping rate and coverage are 98.15% and 99.29%, respectively. The HiFi reads' mapping rate and coverage are 93.66% and 95.91%, respectively. The alignment rate of RNA-seq data is 80.81%. The error rate (homozygous snp ratio) is about 0.02%. The distribution of CG depth indicates there is no apparent contamination in the assembled contigs (Figure 1d). In addition, the assembly has been uploaded to NCBI and passed the NCBI Contamination Screen. All these information indicate that the C. arbutifolia assembly is of high quality and have been added to the text. Please see page 10, line 15.

4. Page 12, line 20-22: What is the biological or evolutionary meaning of the PSGs in the C. arbutifolia genome?
Answer 4: As we submitted the manuscript as Date Note article type, which requires little biological analysis, we did not describe the biological meaning of PSGs. Indeed, among these PSGs, one gene associated with "ATP binding" functions as a growth-regulating factor, and another "integral component of membrane" enriched gene is related to calcium-transporting ATPase. The positive selection of these genes may be related to the characteristics that C. arbutifolia is not easy to form adventitious roots.

5. What is the concrete method of genome collinearity analysis? And what is the statistical data of chromosome fusions and fissions between Salicaceae species, such as related gene numbers or chromosome length?
Answer 5: Syntenic regions between Salicaceae assemblies were based on homology searches carried out by using Mcscan-python (https://github.com/tanghaibao/jcvi/wiki/MCscan-(Python-version)) requiring at least 30 genes per block. We identified 2135, 3351 and 2829 genes in the interchromosomal-recombination regions compared with P. trichocarpa for C. arbutifolia, S. purpurea and S. suchowensis, respectively, which may be related with inter-chromosome fusion and fission events. We have added this statement in Method section, and the detailed information could be found in
Supplementary Table 15 and Supplementary Table 16.

Minor concerns:

1. Page 7, line 8: How did the neutral mutation rate was selected? Is there any published paper which can support it?
Answer 6: Yes, the neutral mutation rate is referred to by Zhou R, et al., which used a mutation rate of 2.5×10^{-9} to estimate the LTR insertion time for Salix purpurea. We have added this reference in the text. Please see page 7, line 13.

Reference:

	Zhou et al. A willow sex chromosome reveals convergent evolution of complex palindromic repeats. Genome Biol. 2020, 21(1):38.<br><br>2. Page 9, line 19: Please provide the Hi-C interaction heatmap. In addition, what is the anchoring rate of contig sequences?<br>Answer 7: The Hi-C interaction heatmap was attached in Figure 2b. A C. arbutifolia genome with a total size of 338.93 Mb was acquired and 95.31% of the assembly sequence was assigned to 19 pseudochromosomes. Please see page 10, line 6.<br><br>3. What is the BUSCO result of the predicted protein sequences?<br>Answer 8: The complete BUSCOs of predicted protein sequences is 97.3%, please see page 11, line 19, and Supplementary Table 8.<br><br>4. Please add the estimated genome size for C. arbutifolia if available.<br>Answer 9: The estimated genome size of C. arbutifolia is 323 Mb. We have added this in the text, please see page 10, line 8.<br><br>5. What is the LAI value of the C. arbutifolia genome?<br>Answer 10: The LTR Assembly Index (LAI) of C. arbutifolia assembly is 10.86. We have added this data in Table 1.<br><br>6. Is there any telomere regions, such as 'CCCTAAA' units which have been broadly discovered in plants, were identified? If so, how many chromosomes have telomere regions.<br>Answer 11: Yes, we have added this analysis using Telomere Identification Toolkit, please see page 10, line 9, and Figure 1e.<br><br>7. Page 13, line 2-7: For the WGD event, please provide the result based on the ks distribution.<br>Answer 12: Yes, we have modified this result using Ks distribution and replaced the relevant figure. Please see page 13, line 19, and Figure 3d.
Additional Information:	
Question	Response
Are you submitting this manuscript to a special series or article collection?	No
**Experimental design and statistics**<br><br>Full details of the experimental design and statistical methods used should be given in the Methods section, as detailed in our Minimum Standards Reporting Checklist. Information essential to interpreting the data presented should be made available in the figure legends.<br><br>Have you included all the information requested in your manuscript?	Yes
**Resources**<br><br>A description of all resources used, including antibodies, cell lines, animals and software tools, with enough	Yes

information to allow them to be uniquely identified, should be included in the Methods section. Authors are strongly encouraged to cite Research Resource Identifiers (RRIDs) for antibodies, model organisms and tools, where possible.<br><br>Have you included the information requested as detailed in our Minimum Standards Reporting Checklist?	
**Availability of data and materials**<br><br>All datasets and code on which the conclusions of the paper rely must be either included in your submission or deposited in publicly available repositories (where available and ethically appropriate), referencing such data using a unique identifier in the references and in the “Availability of Data and Materials” section of your manuscript.<br><br>Have you have met the above requirement as detailed in our Minimum Standards Reporting Checklist?	Yes

Manuscript Click here to access/download;Manuscript;Manuscript-revised Oct 14.doc

The whole-genome assembly of an endangered Salicaceae species: *Chosenia arbutifolia* (Pall.) A. Skv.

Xudong He[1,2,†*], Yu Wang[1,3,†], Jinmin Lian[4,†], Jiwei Zheng[1,2], Jie Zhou[1,2], Jiang Li[4], Zhongyi Jiao[1,2], Yongchao Niu[4], Weiwei Wang[1,2], Jun Zhang[1,2], Baosong Wang[1,2], Qiang Zhuge[3]

[1] Willow Engineering Technology Research Center of National Forestry and Grassland Administration, Jiangsu Academy of Forestry, Nanjing 211153, China

[2] Willow Nursery of the Jiangsu Provincial Platform for Conservation and Utilization of Agricultural Germplasm, Jiangsu Academy of Forestry, Nanjing 211153, China

[3] College of Biology and the Environment, Nanjing Forestry University, Nanjing 210037, China

[4] Biozeron Shenzhen, Inc., Shenzhen 518000, China

[*] Corresponding author: Xudong He

E-mail: hxd_519@163.com; Tel: +86 25 52743830 Fax: +86 25 52741620

[†] These authors contributed equally to this work.

Xudong He [0000-0001-6644-928X];

Jinmin Lian [0000-0002-2063-2086];

Jiwei Zheng [0000-0003-3930-8873];

Jie Zhou [0000-0001-8214-8479];

Jiang Li [0000-0003-2099-8165];

Zhongyi Jiao [0000-0001-6145-8232];

Yongchao Niu [0000-0002-9774-5417];

Qiang Zhuge [0000-0002-9450-487X]

Abstract

Background As a fast-growing tree species, *Chosenia arbutifolia* has a unique, but controversial taxonomic status in the family Salicaceae. Despite its importance as an industrial material, in ecological protection, and in landscaping, *C. arbutifolia* is seriously endangered in Northeast China because of artificial destruction and its low reproductive capability.

Results To clarify its phylogenetic relationships with other Salicaceae species, we assembled a high-quality chromosome-level genome of *C. arbutifoli*a using PacBio HiFi reads and Hi-C sequencing data, with a total size of 338.93 Mb and contig N50 of 1.68 Mb. Repetitive sequences, which accounted for 42.34% of the assembly length, were identified. In total, 33,229 protein-coding genes and 11,474 small ncRNAs were predicted. Phylogenetic analysis suggested that *C. arbutifolia* and poplars diverged approximately 15.3 million years ago, and a large interchromosomal recombination between *C. arbutifolia* and other Salicaceae species was discovered.

Conclusions Our study provides insights into the genome architecture and systematic evolution of *C. arbutifolia*, as well as comprehensive information for germplasm protection and future functional genomic studies.

Keywords *Chosenia arbutifolia*, genome assembly, phylogenetic relationship, genomic comparison

Data Description

Background

As a unique member of the family Salicaceae along with *Populus* and *Salix*, the genus *Chosenia* comprises only one species, *C. arbutifolia* (Pall) A. Skv. (NCBI:txid75699), according to the Flora of China [1]. Compared with poplars and willows, *C. arbutifolia* has several special morphological features, including an unusual leaf shape, extraordinary root system, and particular pistil, stamen, and bract structures [2]. Different from most insect-pollinated *Salix* species, *C. arbutifolia* is wind-pollinated and lacks nectary structures. Therefore, *C. arbutifolia* has been regarded as a transitional species between poplars and willows and treated as an independent genus by some authoritative botanists [3, 4]. However, ample molecular evidence demonstrated that *C. arbutifolia* has a close relationship with *Salix* species and should be considered a member of *Salix* [5]. To date, the taxonomic status of *C. arbutifolia* remains enigmatic and controversial.

C. arbutifolia is mostly distributed along the mountain river banks in Northeast China, and in some areas of the Russian Far East, North Korea, and North Japan [2]. Even beyond the Arctic Circle, *C. arbutifolia* individuals are sporadically found [4]. Owing to its favorable characteristics of strong stress resistance, tremendous shape, and fast growth, *C. arbutifolia* is primarily applicable to industrial materials, ecological protection, and landscape planting. Unlike poplars and willows, *C. arbutifolia* is extremely difficult to propagate using twig cuttings, even when they originate from juvenile individuals [6]. In addition, the natural regeneration of *C. arbutifolia* by means of seed germination requires specific circumstances, including flowing water, an appropriate temperature, and sediment accumulation [4]. In the past decades, the growth area of *C. arbutifolia* has continuously decreased due to excessive deforestation. Furthermore, the species has a weak reproductive capability, resulting in a drastic decline in the natural populations of *C. arbutifolia* and the species has been categorized as endangered in China.

Poplars and willows are expected to serve as novel model systems for genomic and genetic research in woody plants, mainly owing to their dioecism, short growth cycle, easy reproduction, and modest-sized genome [7]. The

accomplishment of whole-genome sequencing of *P. trichocarpa* in 2006 marked a new milestone and paved the way to a poplar genomic research field in the post-genomic era [8]. With the popularization of high-throughput sequencing technologies, numerous other *Populus* species and hybrids have been sequenced and assembled, including *P. euphratica* [9], *P. pruinosa* [10], *P. tremula* and *P. tremuloides* [11], *P. alba* [12], *P. alba* var. *pyramidalis* [13], *P. alba* × *P. tremula* var. *glandulosa* [14], and *P. ilicifolia* [15]. While similar work in the genus *Salix* is slightly lagging behind, an increasing number of whole genome assemblies for the *Salix* species are being reported, including *S. purpurea* (https://phytozome-next.jgi.doe.gov/info/Spurpurea_v5_1), *S. brachista* [16], *S. suchowensis* [17], *S. viminalis* [18], *S. matsudana* [19], and *S. dunnii* [20]. Single-molecule real-time sequencing (SMRT), a third-generation sequencing technology, represents an optimal tool for whole-genome sequencing that overcomes various limits of short-reads sequencing technologies, and has been applied in some important woody plants, including *Liriodendron* [21], *Acer truncatum* [22], *Betula platyphylla* [23], *Paulownia fortune* [24], and *Taxus chinensis* var. *mairei* [25].

Despite its complex taxonomy, essential significance, and endangered status, available genetic and genomic information of *C. arbutifolia* are still scarce. Only a few studies that primarily focused on biological habits, propagation technology, population diversity and protection, phylogenetic analysis, transcriptome sequencing, and gene families are available and were reviewed by He et al. [26]. Here, with the aim to gain a deep insight into the genome architecture of *C. arbutifolia*, we assembled a chromosome-level and highly contiguous genome of *C. arbutifolia* using a combination of SMRT PacBio High-Fidelity (HiFi) reads, Illumina short-read sequencing, and the Hi-C chromosome conformation capture technology. We expected our work to provide substantial genomic resources of *C. arbutifolia* for future functional genomic research on Salicaceae.

Methods

Plant materials and nucleic acid extraction

Branches from a superior individual of *C. arbutifolia* were collected in the town of Manjiang (41°47′10.55″, 127°55′56.13″), Fusong County, Jilin Province, China. All branches were transported back to the laboratory of the Jiangsu Academy of Forestry for hydroponic cultivation until leaves had sprouted. A DNA extraction kit (DP305, Tiangen Biotech, Beijing, China) was used to isolate genomic DNA from young leaves of *C. arbutifolia*. An Omega Plant RNA Kit (Omega Bio-tek, Norcross, GA, USA) was used for total RNA extraction from the leaves of *C. arbutifolia*.

Genome sequencing

According to the standard protocols (Pacific Biosciences, Menlo Park, CA, USA), genomic DNA was fragmented into ~20-kb long reads and used to prepare a PCR-free SMRT bell DNA library, which was sequenced using the circular consensus sequencing mode on the PacBio Sequel platform (PacBio Sequel platform, RRID:SCR_017989). In addition, to generate PE150 short reads, short-insert libraries were constructed using the genomic DNA and then sequenced on the NovaSeq 6000 platform (Illumina NovaSeq 6000 Sequencing System, RRID:SCR_016387) , following the manufacturer's instructions (Illumina, San Diego, CA, USA).

Hi-C sequencing

The Dovetail Hi-C library preparation kit (Dovetail Genomics, Scotts Vally, CA, USA) was used for Hi-C library construction, according to the manufacturer's instructions. Briefly, formaldehyde was used to fix the nuclear chromatin. After extraction, the restriction enzyme, *Dpn*-II was selected for digestion. Biotinylated nucleotides were filled and ligated to the sticky ends. After the revision of the crosslinks, free biotin was eliminated from the ligated fragments. The DNA was purified and sheared to ~350 bp. Via streptavidin bead pulldown, biotinylated fragments were enriched and amplified by PCR for library construction. The library was sequenced on the Illumina NovaSeq

platform.

Genome assembly

The software DipAsm [27] was employed to construct contigs of *C. arbutifolia* using the Pacbio HiFi reads to generate a haplotype-resolved assembly. The detailed parameters and settings used for DipAsm assembly were described in Supplementary Table S1. Then, the raw contigs were polished in two rounds based on the short reads generated by Illumina sequencing using the program Pilon v1.22 (Pilon, RRID:SCR_014731) [28].

Hi-C scaffolding

Hi-C technology was utilized to assist in the initial assembly to generate a chromosome-scale genome of *C. arbutifolia*. First, to filter the raw Hi-C reads, the program Hic-Pro v2.11.1 (Hic-Pro, RRID:SCR_017643) [29] was used to map the Illumina short reads onto the polished temporary genome with the default parameters. Then, invalid, non-ligated, and self-ligated reads were discarded. Subsequently, the genomic contigs were clustered into potential chromosomal groups using the software Juicer v1.6.2 (Juicer, RRID:SCR_017226) [30] and 3d-DNA v180114 (3D de novo assembly, RRID:SCR_017227) [31]. Next, the contig orientation was validated using the assembly tool JuiceBox v1.11.8 (Juicebox, RRID:SCR_021172) [30] and the ambiguous fragments were removed manually. Finally, the completeness of the genome assembly was evaluated using the software BUSCO v5.2.1 (BUSCO, RRID:SCR_015008) [32]. The "CCCTAAA" telomeric repeat in the *C. arbutifolia* assembly was identified using Telomere Identification Toolkit.

Characterization of repetitive sequences

The *C. arbutifolia* genome was screened for tandem and interspersed repeats. The software Tandem Repeats Finder v4.07 [33] was used to identify the tandem repeat contents. For the identification of interspersed repetitive sequences,

a strategy combining *de novo* and given repeat searching was performed. The tools RepeatModeler v1.0.8 (RepeatModeler, RRID:SCR_015027) and LTR_FINDER v1.0.6 (LTR_Finder, RRID:SCR_015247) [34] were employed for the prediction of *de novo* repeat sequences. Then, RepeatMasker v4.0.7 (RepeatMasker, RRID:SCR_012954) was employed to screen the *C. arbutifolia* genome against the combined *de novo* transposable element library. RepeatMasker v4.0.7 and the Repbase database (Repbase, RRID:SCR_021169) [35] were used to identify known transposable element repeats.

LTR insertion time estimation

The program LTR_FINDER v1.06 [34] was applied to detect LTRs in the *C. arbutifolia* genome to estimate insertion times, with parameter settings '-D 15000 -d 1000 -L 7000 -l 100 -p 20 -C -M 0.9'. Then, using the LTR_retriever (RRID:SCR_017623) pipeline, the results were integrated, and the false positives were removed from the primitive predictions. The insertion time was calculated as $T = K / 2r$, where K and r represent the divergence rate and neutral mutation rate ($r = 2.5 \times 10^{-9}$), respectively [36].

Genome annotation

The protein sequences of seven plant genomes, including *Manihot esculenta*, *Linum usitatissimum*, *S. purpurea*, *P. trichocarpa*, *R. communis*, *Jatropha curcas*, and *Arabidopsis thaliana*, were accessed from the NCBI and Phytozome database and mapped to the assembled genome of *C. arbutifolia* using the software genBlastA v1.0.4 (genBlastA, RRID:SCR_020951) [37]. Based on each genBlastA hit, the software GeneWise v2.4.1 (GeneWise, RRID:SCR_015054) [38] was employed to predict the exact gene structure. Three programs for *de novo* gene prediction, Augustus v3.2.1 (Augustus, RRID:SCR_008417)[39], GlimmerHMM v3.0.4 (GlimmerHMM, RRID:SCR_002654) [40], and SNAP v2006-07-28 (SNAP, RRID:SCR_002127) [41], were applied to explore coding regions in the assembly of *C. arbutifolia*. The software HISAT2 v2.0.1 (HISAT2, RRID:SCR_015530) [42]

was used to map RNA-seq data to the chromosome-scaled *C. arbutifolia* assembly, and then, StringTie v1.2.2 (StringTie, RRID:SCR_016323) [43] was used to assemble the transcripts. The program TransDecoder v3.0.1 (TransDecoder, RRID:SCR_017647) was conducted to identify the candidate coding regions. Using the above approaches, all predicted gene models were integrated by EvidenceModeler (EVidenceModeler, RRID:SCR_014659) [44] into a non-redundant set of gene structures that were finally refined with the Program to Assemble Spliced Alignments (PASA) v2.3.3 (PASA, RRID:SCR_014656) [45]. The protein-coding genes were functionally annotated against two integrated SwissProt and TrEMBL databases using BLASTP (BLASTP, RRID:SCR_001010) [46] with E-value 1e-05. The software InterProScan v5.30 (InterProScan, RRID:SCR_005829)[47] was employed for protein domain annotation. For all genes, the BLAST GO terms were extracted using InterProScan v5.30) and the pathways were assigned against the KEGG database (release 84.0) using BLAST (NCBI BLAST, RRID:SCR_004870) .

Non-coding RNA prediction

Non-coding RNAs, including four types of transfer RNAs (tRNAs), ribosomal RNAs (rRNAs), small nuclear RNAs (snRNAs), and micro-RNAs (miRNAs), were predicted. tRNAs and rRNAs were discovered using tRNAscan-SE v1.3.1 (tRNAscan-SE, RRID:SCR_010835) [48] and BLASTn v2.2.24 (BLASTN, RRID:SCR_001598, E-value 1e-5) via the alignment to template rRNA and tRNA sequences of *Oryza* and *Arabidopsis*, respectively. SnRNAs and miRNAs were screened from the Rfam database (Rfam, RRID:SCR_007891) using INFERNAL v1.1.1 (Infernal, RRID:SCR_011809) .

Gene family analysis

The OrthoMCL v2.0.9 (OrthoMCL, RRID:SCR_007839) [49] clustering program was run on the proteomes of *C. arbutifolia*, *S. purpurea*, *S. suchowensis*, *S. viminalis*, *S. brachista*, *P. euphratica*, *P. tremuloides*, *P. tremula*, *P. pruinosa*, *P. trichocarpa*, *P. alba*, and *R. communis*. A phylogenetic tree for these 12 species was constructed using

the identified single-copy gene families. From each family, four-fold degenerate sites were segregated and concatenated into one supergene. The phylogenetic tree was reestablished using the program MrBayes v3.1.2 (MrBayes, RRID:SCR_012067) with the model of GTR + gamma substitution. The program MCMCtree v4.4 in the PAML package (PAML, RRID:SCR_014932) [50] was used to estimate the divergence times among the 12 species, with the JC69 nucleotide substitution model and an independent rates clock. The calibration divergence times between *S. purpurea* and *P. trichocarpa* (~35.6 MYA), and *R. communis* and *P. trichocarpa* (~80 MYA) were obtained from the TimeTree database (TimeTree, RRID:SCR_021162) [51]. Changes in gene family size within the phylogenetic tree were analyzed using CAFE v2.1 (CAFE, RRID:SCR_005983) [52]. Positive selection genes in the *C. arbutifolia* genome were detected using the branch-site model incorporated in the PAML package (RRID:SCR_014932) [50] and a maximum likelihood ratio test based on the single copy genes. *C. arbutifolia* and the other 11 species (except *R. communis*) were determined as foreground and background branches of the phylogenetic tree, respectively. GO enrichment was derived using Fisher's exact test followed by Benjamini-Hochberg adjustments, with the cutoff of $P < 0.05$. WGD events were inferred based on the distribution of distance-transversion rate at 4DTv of paralogous gene pairs. The 4DTv transversion rates between all species pairs were calculated using an in-house Perl script.

Synteny analysis

Syntenic regions between Salicaceae assemblies were based on homology searches carried out by conducting with Mcscan (MCScan, RRID:SCR_017650) (Python-version)] requiring at least 30 genes per block.

Results and Discussion

Genome assembly

In total, 34.22 Gb with a ~101× HiFi read coverage were generated through whole-genome sequencing of *C.*

arbutifolia using the PacBio Sequel platform (Fig. 1a, Supplementary Table S2). The PacBio reads were assembled and polished with ~111× Illumina paired-end reads (37.52 Gb, Supplementary Table S3), resulting in ~1.68 Mb of contig N50 (Table 1). Subsequently, another 27.81 Gb Dovetail Hi-C data with a ~82× depth were utilized to refine the genome assembly (Supplementary Table S3). Thus, a *C. arbutifolia* genome with a total size of 338.93 Mb was acquired and 95.31% of the assembly sequence was assigned to 19 pseudochromosomes (Fig. 1b), which is close to the estimated genome size (323 Mb, Fig. 1c) and similar to those of *S. dunnii* [20] (328 Mb), *S. purpurea* (329.29 Mb, Table 1) and *S. brachista* [16] (339.58 Mb), but slightly smaller than those of *suchowensis* [17] (356.5Mb) and *S. viminalis* [18] (357.06 Mb). Compared with the genome sizes of *Populus* species, such as *P. trichocarpa* [8] (434.13 Mb), *P. euphratica* [9] (496.5Mb), *P. pruinosa* [10] (479.3 Mb), *P. tremula* [11] (390 Mb), *P. tremuloides* [11] (378 Mb), *P. alba* [12] (415.99 Mb), *P. alba* var. *pyramidalis* [13] (464 Mb), and *P. ilicifolia* [15] (402 Mb), those of *C. arbutifolia* and *Salix* species are generally substantially smaller, which is consistent with early reports [17, 53]. The super-scaffolds number, super-scaffold N50, and maximum super-scaffold length were 304, ~16.46 Mb, and 31.95 Mb, respectively (Table 1). To evaluate the assembly quality of the *C. arbutifolia* genome, we mapped the next-generation sequencing short reads to the assembly, getting a mapping rate and coverage of 98.15% and 99.29%, respectively. The distribution of CG depth indicated there was no apparent contamination in the assembled sequences (Fig. 1d). A telomere unit 'CCCTAAA' that broadly discovered in plants was also detected in most assembled persudo-chromosome sequences, except chromosome four, five, six, seven, seventeen, and eighteen (Supplementary Fig. 1e). In addition, 1591 core genes were identified in the OrthoDB embryophyta database, accounting for 98.6% of the total 1614 core genes, among which single-copy and duplicated genes represented 87.0% and 11.6%, respectively (Supplementary Table S4). The assembled *C. arbutifolia* genome and the features of different Salicaceae species are illustrated in Figure 2a and Table 1, respectively.

Repetitive sequence identification

Among the assembled genome sequences of *C. arbutifolia*, a total of ~143.47 Mb (42.34%) repeat element sequences

were screened, of which tandem and interspersed repeats accounted for 8.47% and 38.21%, respectively (Supplementary Table S5). Among the interspersed repeats, three types of repetitive elements, including Class I (retrotransposons), Class II (DNA transposons), and unclassified elements, representing 38.21% of the genome assembly, were identified (Supplementary Table S6). The long terminal repeat (LTR) retrotransposons represented the most frequent among Class I repetitive sequences, with Gypsy and Copia LTR retrotransposons accounting for 14.90% and 14.25%, respectively, whereas long and short interspersed nuclear elements represented approximately 3% of the genome size. The insertion time of LTR retrotransposons was predicted by detecting the sequence divergence at both ends of impact LTRs. As shown in Fig. 2b, a surge of retrotransposon amplification was detected in *C. arbutifolia* approximately 0.472 million years ago (MYA), indicating an expansion event in the recent period of genome evolution.

Gene annotation

Through a combined prediction strategy of *ab initio*, homologous protein, and transcriptome, 33,229 protein-coding genes were predicted (Supplementary Table S6). Of these, 31,618 (95.15%) were successfully annotated in diverse databases, including NCBI nr, Swissprot, KEGG, TrEMBL, and InterPro, whereas the remaining 1611 (4.85%) genes had no significant correspondence with sequences in public databases (Supplementary Table S7, Fig. 2c). In addition, the complete BUSCOs of the predicted proteins is about 97.3% (Supplementary Table S8), indicating the high quality of the annotated genes. The reason for the overall smaller genomes of the *Salix* species has been suggested to be the faster evolution speed of willows, which reduces the predicted gene number [17, 53]. However, we found that there is no linear correspondence between the genome size and the number of predicted genes in the Salicaceae species. For example, 36,490 genes have been identified in *S. viminalis* [18] in an assembled genome of 357.06 Mb. The *S. viminalis* genome is smaller in size but harbors a larger number of genes than those of *P. pruinosa* [10] (35,131 genes), *P. alba* [12] (32,963 genes) and *P. ilicifolia* [15] (33,684 genes). *S. brachista* [16] has a slightly larger

genome (339.58 Mb), but smaller number of predicted genes (30,209) than *C. arbutifolia* (338.93 Mb; 33,229 genes) and *S. purpurea* (329.29 Mb; 35,125 genes). Undoubtedly, the efficiency of genome assembly and the strategy used for gene mining are essential factors affecting the numbers of predicted genes in different species. The mean length of the predicted protein-coding genes was 3156 bp, with 5.02 exons per gene, and the average lengths of exons and introns were 233 bp and 446 bp, respectively (Supplementary Table S9). Non-coding RNAs in the *C. arbutifolia* genome were explored and annotated, and comprised 239 miRNAs, 697 tRNAs 10,043 rRNAs, and 495 snRNAs (Supplementary Table S10).

Phylogenetic relationship analysis

The protein-coding genes of 11 Malpighiales species, including *S. purpurea*, *S.suchowensis*, *S. viminalis*, *S. brachista*, *P. trichocarpa*, *P. tremuloides*, *P. tremula*, *P. pruinosa*, *P. euphratica*, *P. alba*, and *Ricinus communis*, were collected from relevant databases and clustered into 30,618 gene families together with the protein-coding genes of *C. arbutifolia* (Supplementary Table S11, Fig. 3a). The analysis of gene family intersection exhibited that 11,308 gene families were shared by the 11 Salicaceae species, but not *R. communis* (Fig. 3b). For *C. arbutifolia*, 28,512 genes were assigned to 18,729 genes families, of which 184 families, containing 1,750 genes in total, were specific when compared with the 11 other Malpighiales species (Supplementary Table S11). These genes were significantly enriched in the Gene Ontology (GO) terms “DNA binding”, “ribonucleoside binding”, and “DNA-directed 5'-3' RNA polymerase activity” with FDR < 0.05 (Supplementary Table S12).

A phylogenetic tree was constructed for the 12 Malpighiales species, considering *R. communis* as an outgroup (Fig. 3c). The divergence time between *Chosenia* and *Populus* was assessed to be around 15.3 MYA, and *C. arbutifolia* was separated from the four *Salix* species around ~6.6 MYA, indicating that *C. arbutifolia* was the first species to differentiate from *Populus* and may be a transitional species between poplars and willows. It preserved some poplar characteristics, such as the haploid number (n = 19, most of the tree species in *Salix* are polyploid), wind pollination,

and absence of glands. In addition, previous reports on *S. brachista* [16] and *S. dunnii* [20] demonstrate exactly the same relationships and similar divergence times among the above-mentioned species, indicating that *S. suchowensis* may have evolved substantially further than other *Salix* species owing to a stronger purifying selection [17, 53]. However, the family Salicaceae comprises more than 600 species worldwide, and limited available genome data of Salicaceae species were analyzed in this study. Thus, to completely clarify the phylogenetic history of this family, more species should be added in the future.

Compared with the most recent common ancestor (MRCA) of *Chosenia* and *Salix, C. arbutifolia* showed 72 and 85 expansion and contraction events of each gene family, respectively (Fig. 3c). The results of GO enrichment analysis revealed that among the expanded genes, 29 genes were associated with “heme binding” and “oxidation-reduction process”, and 22 genes were involved in “iron ion binding” (Supplementary Table S13). Among the contracted genes, 93 genes were related to “ATP binding”, and 88 genes were responsible for “protein kinase activity” and “protein phosphorylation” (Supplementary Table S13). Positive selection genes (PSGs) were detected using single-copy gene sets of the 12 species. In *C. arbutifolia*, a total of 89 PSGs were detected, of which six and five PSGs were enriched in the GO terms of “integral component of membrane” and “catalytic activity”, respectively, whereas three PSGs were related to both “ATP binding” and “nucleic acid binding” (Supplementary Table S14).

Whole-genome duplication analysis

Whole genome duplication (WGD) events were deduced by examining distributions of synonymous substitutions per site (Ks) within the *C. arbutifolia* genome. After the speciation between *C. arbutifolia* and *Arabidopsis thaliana* (Ks = 3.47), a common salicoid WGD event occurred (Ks = 0.36). The divergence between *C. arbutifolia* and *P. trichocarpa* emerged at the peak of Ks ~ 0.13, followed by *C. arbutifolia* and *S. purpurea* (Ks = 0.06), which is in consistent with the results of phylogenetic analysis (Fig. 3d). After the differentiation of the Salicaceae species, there was no obvious evidence of a *C. arbutifolia*-specific WGD.

Genome collinearity analysis

The genome collinearity among *C. arbutifolia*, *S. purpurea*, *S. suchowensis*, and *P. trichocarpa* was analyzed. The syntenic regions showed that most chromosomes were highly conserved among the Salicaceae species, except for a large interchromosomal-recombination between chromosomes one and sixteen (Fig. 4a). Compared with *P. trichocarpa*, we have identified 2135, 3351 and 2829 genes in the whole genome interchromosomal-recombination regions for *C.arbutifolia*, *S.purpurea* and *S.suchowensis*, respectively (Supplementary Table S15, Supplementary Table S16). Furthermore, the whole chromosomes of *C. arbutifolia* and the two *Salix* species were highly collinear (Fig. 4b). Together, these results indicated that main chromosomal fissions and fusions have occurred during the evolution of Salicaceae, resulting in a genera divergence of Salicaceae. Like in other *Salix* species, such as *S. brachista* [16], *S. suchowensis* [17, 53], and *S. dunnii* [20], most of the chromosomes of *C. arbutifolia* were highly conserved with *P. trichocarpa*, except for chromosomes one and sixteen, where a large interchromosomal recombination was discovered (Fig. 4a). It has been reported that the chromosomal fusions and fissions that emerged in *Populus* after a lineage-specific salicoid duplication gave rise to the divergence of the two genera, *Populus* and *Salix* [54]. Nevertheless, recombination modes are quite different between *S. suchowensis* and other *Salix* species. Chromosome sixteen of *S. suchowensis* entirely originated from a partial chromosome one of *P. trichocarpa*, and chromosome 1 of *S. suchowensis* was comprised of the remaining part of chromosome one and the entire chromosome sixteen of *P. trichocarpa* [17]. However, in our study, chromosome sixteen of *C. arbutifolia* was fused with a partial chromosome one and the entire chromosome sixteen of *P. trichocarpa*, and chromosome one of *C. arbutifolia* comprised the remaining part of *P. trichocarpa* chromosome one. This difference was confirmed by collinearity analysis (Fig. 4b) and the same phenomenon was also detected in *S. brachista* [16] and *S. dunnii* [20].

Conclusions

Although multiple genomes of *Populus* and *Salix* species have been reported, we sequenced and assembled a genome

of the taxonomically difficult species *C. arbutifolia* that belongs to the monotypic genus *Chosenia* for the first time by using PacBio HiFi reads, Hi-C chromatin contact maps, and Illumina short reads. As a significant supplementary for the family Salicaceae, the assembled genome shed a deep insight into the genomic architecture of *C. arbutifolia* and revealed the systematic evolution and phylogenetic relationships with other Salicaceae species. Given the limited genomic resources in the public databases, it is worthwhile to take full advantage of more available genomic information for further studies. Overall, our results lay a solid foundation for genetic and genomic research on Salicaceae species in future. All supporting data and materials are available in the GigaScience GigaDB database [55].

Data Availability

The genome assembly and all the sequencing data have been deposited in the GenBank database under the accession number PRJNA788330. All supporting data and materials are available in the *GigaScience* GigaDB database [55].

Additional Files

Supplementary table S1. The parameters and settings used for DipAsm assembly

Supplementary Table S2. Statistics of Pacbio HiFi data

Supplementary Table S3. Statistics of Illumina data

Supplementary Table S4. Statistics of the *C. arbutifolia* assembly gene-space with the 1440 BUSCO embryophyta gene set

Supplementary Table S5. General statistics of the repeats in *C. arbutifolia* genome

Supplementary Table S6. Interspersed repeats (TEs) content in the assembled *C. arbutifolia* genome

Supplementary Table S7. Functional annotation of the predicted genes for *C. arbutifolia*

Supplementary Table S8. Statistics of the predicted protein-coding genes in different species

Supplementary Table S9. Statistic of the predicted proteins with the 1614 BUSCO embryophyta gene set

Supplementary Table S10. Non-coding RNAs in the *C. arbutifolia* genome

Supplementary Table S11. Statistics of gene families of the twelve Malpighiales species

Supplementary Table S12. GO enrichment of the specific genes in *C. arbutifolia*

Supplementary Table S13. GO enrichment of expanded and contracted genes in *C. arbutifolia*

Supplementary Table S14. GO enrichment of positive selection genes in *C. arbutifolia*

Supplementary Table S15. The interchromosomal-recombination blocks between Salicaceae species

Supplementary Table S16. Statistic of the gene numbers in interchromosomal-recombination regions between Salicaceae species.

Abbreviations

4DTv: 4-fold degenerate sites; BLAST: Basic Local Alignment Search Tool; BUSCO: Benchmarking Universal Single-Copy Orthologs; GO: Gene Ontology; HiFi: High-Fidelity; KEGG: Kyoto Encyclopedia of Genes and Genomes; LTR: long terminal repeat; miRNAs: micro-RNAs; MRCA: most recent common ancestor; MYA: million years ago; PSGs: positive selection genes; RLKs: receptor-like kinases; rRNAs: ribosomal RNAs; RNA-seq: RNA sequencing; PASA: Program to Assemble Spliced Alignments; SMRT: Single-Molecule Real-time Sequencing; snRNAs: small nuclear RNAs; tRNAs: transfer RNAs; WGD: whole-genome duplication.

Funding

This work was financially supported by the National Natural Science Foundation of China (Grant No. 31670662) and the Independent Scientific Research Project of Jiangsu Academy of Forestry (Grant No. ZZKY202101).

Competing Interests

The authors declare that they have no conflict of interest.

Authors' Contribution

XDH and QZ conceived and designed the experiments. XDH wrote and revised the manuscript. YW, JML, JWZ, JZ, JL, ZYJ and YCN analyzed the data. BSW and WWW collected the samples. JZ processed the data. All authors have read and approved the final manuscript.

Acknowledgements

The authors are grateful to Jun Ren in Jilin Academy of Forestry for their assistance with sample collection. Special thanks to Prof. Tongming Yin, Siming Gan and reviewers for their valuable comments on the manuscript.

References

1. Wang Z, Fang CF. Salicaceae. In Flora Republicae Popularis Sinicae. Science Press; 1984. p. 79-81.

2. Kadis I. Chosenia: an amazing tree of Northeast Asia. *Arnoldia*. 2005;**63**(3):8-17.

3. Nakai T. *Chosenia*, a new genus of Salicaceae. *Bot Mag.* 1920;**34**:66-9.

4. Moskalyuk TA. *Chosenia arbutifolia* (Salicaceae): life strategies and introduction perspectives. *Siberian J For Sci.* 2016;**3**:34-45.

5. He XD, Wang Y, Zheng JW, *et al*. Phylogenetic analysis of *Chosenia arbutifolia* (Pall.) A. Skv. in Salicaceae using complete chloroplast genome sequence. *Ann For Res.* 2022;**65**(1): 3-16.

6. Tu ZY. Breeding and cultivation of *Salix*. Jiangsu Science and Technology Press; 1982. p. 154-196.

7. Hanley S, Mallott M, Karp A. Alignment of a *Salix* linkage map to the *Populus* genomic sequence reveals macrosynteny between willow and poplar genomes. *Tree Genet Genomes.* 2006;**3**(1): 35-48.

8. Tuskan GA, DiFazio S, Jansson S, *et al*. The genome of black cottonwood, *Populus trichocarpa* (Torr. & Gray). *Science.* 2003;**313**(5793):1596-1604.

9. Ma T, Wang JY, Zhou GK, *et al*. Genomic insights into salt adaptation in a desert poplar. *Nat Commun.* 2013;**4**(1):2797.

10. Yang WL, Wang K, Zhang J, *et al*. The draft genome sequence of a desert tree *Populus pruinosa*. *GigaScience.* 2017;**6**(9):1-7.

11. Lin YC, Wang J, Delhomme N, *et al*. Functional and evolutionary genomic inferences in *Populus* through genome and population sequencing of American and European aspen. *PNAS.* 2018;**115**(46):E10970-8.

12. Liu YJ, Wang XR, Zeng QY. *De novo* assembly of white poplar genome and genetic diversity of white poplar population in Irtysh River basin in China. *Sci China Life Sci.* 2019;**62**(5): 609-18.

13. Ma JC, Wan DS, Duan BB, *et al*. Genome sequence and genetic transformation of a widely distributed and cultivated poplar. *Plant Biotechnol J.* 2019;**17**(2):451-60.

14. Qiu DY, Bai SL, Ma JC, *et al*. The genome of *Populus alba* × *Populus tremula* var. *glandulosa* clone 84K. *DNA Res.* 2019;**26**(5):423-31.

15. Chen ZY, Ai FD, Zhang JL, *et al*. Survival in the Tropics despite isolation, inbreeding and asexual reproduction: insights from the genome of the world's southernmost poplar (*Populus ilicifolia*). *Plant J.* 2020;**103**(1):430-42.

16. Chen JH, Huang Y, Brachi B, *et al*. Genome-wide analysis of cushion willow provides insights into alpine plant divergence in a biodiversity hotspot. *Nat Commun.* 2019;**10**(1): 5230.

17. Wei SY, Yang YH, Yin TM. The chromosome-scale assembly of the willow genome provides insight into

Salicaceae genome evolution. *Horticul Res.* 2020;**7**:45.

18. Almeida P, Proux-Wera E, Churcher A, *et al*. Genome assembly of the basket willow, *Salix viminalis*, reveals earliest stages of sex chromosome expansion. *BMC Biol.* 2020;**18**: 78.

19. Zhang J, Yuan HW, Li YJ, *et al*. Genome sequencing and phylogenetic analysis of allotetraploid *Salix matsudana* Koidz. *Horticul Res.* 2020;**7**:201.

20. He L, Jia KH, Zhang RG, *et al*. Chromosome-scale assembly of the genome of *Salix dunnii* reveals a male-heterogametic sex determination system on chromosome 7. *Mol Ecol Resour.* 2021;**21**(6):1966-82.

21. Chen JH, Hao ZD, Guang XM, *et al*. Liriodendron genome sheds light on angiosperm phylogeny and species-pair differentiation. *Nat Plants.* 2019;**5**:18-25.

22. Ma QY, Sun TL, Li SS, *et al*. The *Acer truncatum* genome provides insights into nervonic acid biosynthesis. *Plant J.* 2020;**104**(3):662-78.

23. Chen S, Wang YC, Yu LL, *et al*. Genome sequence and evolution of *Betula platyphylla*. *Hortic Res.* 2021;**8**:37.

24. Cao YB, Sun GL, Zhai XQ, *et al*. Genomic insights into the fast growth of paulownias and the formation of *Paulownia* witches' broom. *Mol Plant.* 2021;**14**(10):1668-82.

25. Xiong XY, Gou JB, Liao QG, *et al*. The *Taxus* genome provides insights into paclitaxel biosynthesis. *Nat Plants.* 2021;**7**:1026-36.

26. He XD, Wang Y, Zheng JW, *et al*. Full-length transcriptome characterization and comparative analysis of *Chosenia arbutifolia*. *Forests.* 2022;**13**(4):543.

27. Garg S, Fungtammasan A, Carroll A, *et al*. Chromosome-scale, haplotype-resolved assembly of human genomes. *Nat Biotechnol.* 2021;**39**(3):309-12.

28. Walker BJ, Abeel T, Shea T, *et al*. Pilon: an integrated tool for comprehensive microbial variant detection and genome assembly improvement. *PLoS ONE.* 2014;**9**(11):e112963.

29. Servant N, Varoquaux N, Lajoie BR, *et al*. HiC-Pro: an optimized and flexible pipeline for Hi-C data processing. *Genome Biol.* 2015;**16**:259.

30. Durand NC, Shamim MS, Machol I, *et al*. Juicer provides a one-click system for analyzing loop-resolution Hi-C experiments. *Cell Syst.* 2016;**3**(1):95-8.

31. Dudchenko O, Batra SS, Omer AD, *et al*. *De novo* assembly of the *Aedes aegypti* genome using Hi-C yields chromosome-length scaffolds. *Science.* 2017;**356**(6333):92-5.

32. Simão FA, Waterhouse RM, Ioannidis P, *et al*. BUSCO: assessing genome assembly and annotation completeness with single-copy orthologs. *Bioinformatics.* 2015;**31**(19): 3210-12.

33. Benson G. Tandem repeats finder: a program to analyze DNA sequences. *Nucleic Acids Res.* 1999;**27**(2):573-80.

34. Xu Z, Wang H. LTR_FINDER: an efficient tool for the prediction of full-length LTR retrotransposons. *Nucleic Acids Res.* 2007;**35**(suppl_2):W265-8.

35. Bao WD, Kojima KK, Kohany O. Repbase Update, a database of repetitive elements in eukaryotic genomes. *Mobile DNA.* 2015;**6**:11.

36. Zhou R, Macaya-Sanz D, Carlson CH, *et al.* A willow sex chromosome reveals convergent evolution of complex palindromic repeats. *Genome Biol.* 2020;**21**(1):38.

37. She R, Chu JS, Wang K, *et al.* genBlastA: enabling BLAST to identify homologous gene sequences. *Genome Res.* 2008;**19**(1):143-9.

38. Birney E, Clamp M, Durbin R. GeneWise and Genomewise. *Genome Res.* 2004;**14**(5):988-95.

39. Stanke M, Keller O, Gunduz I, *et al.* AUGUSTUS: *ab initio* prediction of alternative transcripts. *Nucleic Acids Res.* 2006;**34**(suppl_2):W435-9.

40. Majoros WH, Pertea M, Salzberg SL. TigrScan and GlimmerHMM: two open source *ab initio* eukaryotic gene-finders. *Bioinformatics.* 2004;**20**(16):2878-9.

41. Korf I. Gene finding in novel genomes. *BMC Bioinformatics.* 2004; **5**:59.

42. Kim D, Langmead B, Salzberg SL. HISAT: a fast spliced aligner with low memory requirements. *Nat Methods.* 2015;**12**(4):357-60.

43. Kovaka S, Zimin AV, Pertea GM, *et al.* Transcriptome assembly from long-read RNA-seq alignments with StringTie2. *Genome Biol.* 2019;**20**(1):278.

44. Haas BJ, Salzberg SL, Zhu W, *et al.* Automated eukaryotic gene structure annotation using EVidenceModeler and the program to assemble spliced alignments. *Genome Biol.* 2008;**9**:R7.

45. Haas BJ, Delcher AL, Mount SM, *et al.* Improving the Arabidopsis genome annotation using maximal transcript alignment assemblies. *Nucleic Acids Res.* 2003;**31**(19):5654-66.

46. McGinnis S, Madden TL. BLAST: at the core of a powerful and diverse set of sequence analysis tools. *Nucleic Acids Res.* 2004;**32**(suppl_2):W20-5.

47. Quevillon E, Silventoinen V, Pillai S, *et al.* InterProScan: protein domains identifier. *Nucleic Acids Res.* 2005;**33**(suppl_2):W116-20.

48. Lowe TM, Eddy SR. tRNAscan-SE: a program for improved detection of transfer RNA genes in genomic sequence. *Nucleic Acids Res.* 1997;**25**(5):955-64.

49. Li L, Stoeckert CJ, Roos DS. OrthoMCL: identification of ortholog groups for eukaryotic genomes. *Genome Res.*

2003;**13**(9):2178-89.

50. Yang ZH. PAML: a program package for phylogenetic analysis by maximum likelihood. *Bioinformatics.* 1997;**13**(5):555-6.

51. Hedges SB, Dudley J, Kumar S. TimeTree: a public knowledge-base of divergence times among organisms. *Bioinformatics.* 2006;**22**(23):2971-2.

52. Bie TD, Cristianini N, Demuth JP, *et al*. CAFE: a computational tool for the study of gene family evolution. *Bioinformatics.* 2006;**22**(10):1269-71.

53. Hou J, Wei SY, Pan HX, *et al*. Uneven selection pressure accelerating divergence of *Populus* and *Salix*. *Horticul Res.* 2019;**6**:37.

54. Hou J, Ye N, Dong ZY, *et al*. Major chromosomal rearrangements distinguish willow and poplar after the ancestral "Salicoid" genome duplication. *Genome Biol Evol.* 2016;**8**(6): 1868-75.

55. He X, Wang Y, Lian J, *et al*. Supporting data for "The whole-genome assembly of an endangered Salicaceae species: Chosenia arbutifolia (Pall.) A. Skv." *GigaScience Database*. 2022. http://doi.org/10.5524/102329

Table 1: Comparison of Salicaceae assemblies

Assembly feature	*C. arbutifolia*	*S. suchowensis*	*S. purpurea*	*P. trichocarpa*
Size (Mb)	338.93	356.5	329.29	434.13
No. of super-scaffolds	304	1,201	348	1,446
Contig N50 (bp)	1,682,645	263,908	5,083,238	552,806
Super-scaffold N50	16,460,042	16,776,717	14,688,223	19,465,461
Longest super-scaffold (Mb)	31.95	34.98	32.43	50.50
No. of protein coding genes	33,229	36,937	35,125	41,335
Complete BUSCOs (%)	98.6	97.3	98.4	98.8
LTR assembly index	10.86	17.06	13.82	8.15

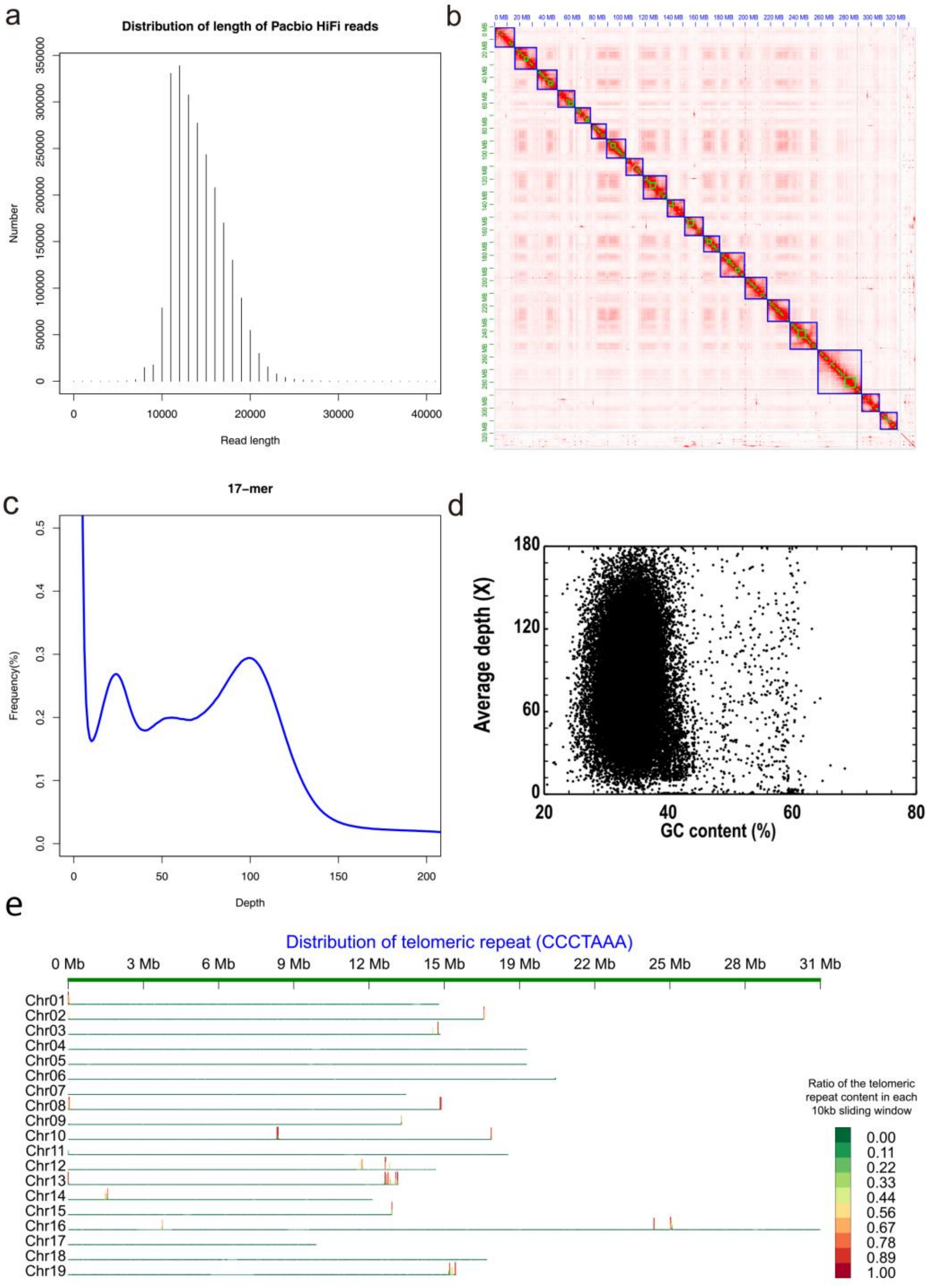

Figure 1: Genome sequencing and assembly of *C. arbutifolia*. **a,** The length distribution of Pacbio HiFi reads. X-axis: the length of HiFi reads; Y-axis: the number of HiFi reads at a given length. **b,** The Hi-C interaction heatmap for genome-wide analysis of chromatin interactions in the *C. arbutifolia* genome. **c**, The 17-mer distribution of Illumina data. X-axis: the sequencing depth; Y-axis: the proportion of a K-mer at a given sequencing depth. **d,** The relationship between GC content and sequencing depths base on the alignment of Pacbio HiFi data. **e,** The distribution of 'CCCTAAA' telomeric repeat in the nineteen chromosomes of *C. arbutifolia* assembly.

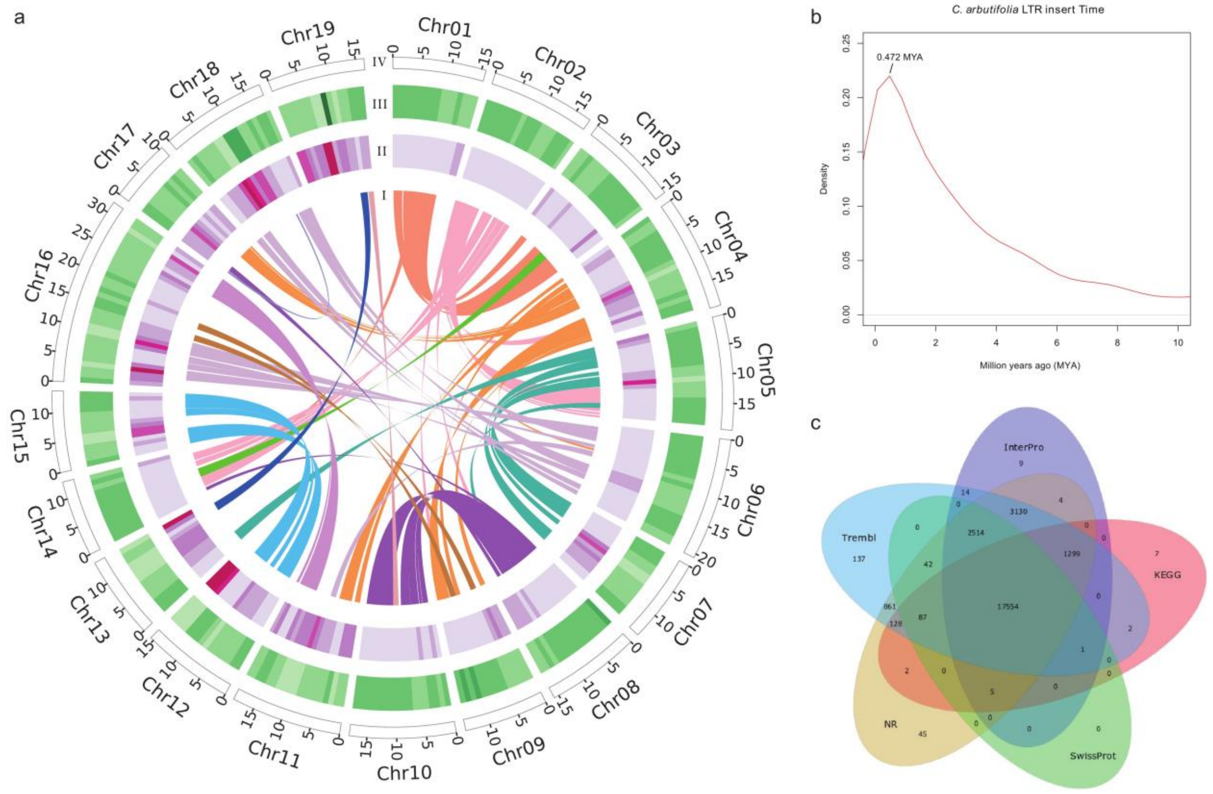

Figure 2: *C. arbutifolia* genome characeristics. **a,** Genome circos plot. I: Collinear regions within the *C. arbutifolia* assembly; II: Percentage of transposable elements in 1 Mb sliding windows; III: Gene density in 1Mb sliding windows; IV: Chromosomes length in Mb. **b,** Insertion times of LTR retrotransposons. **c,** Venn diagram showing genes shared among different annotated datasets.

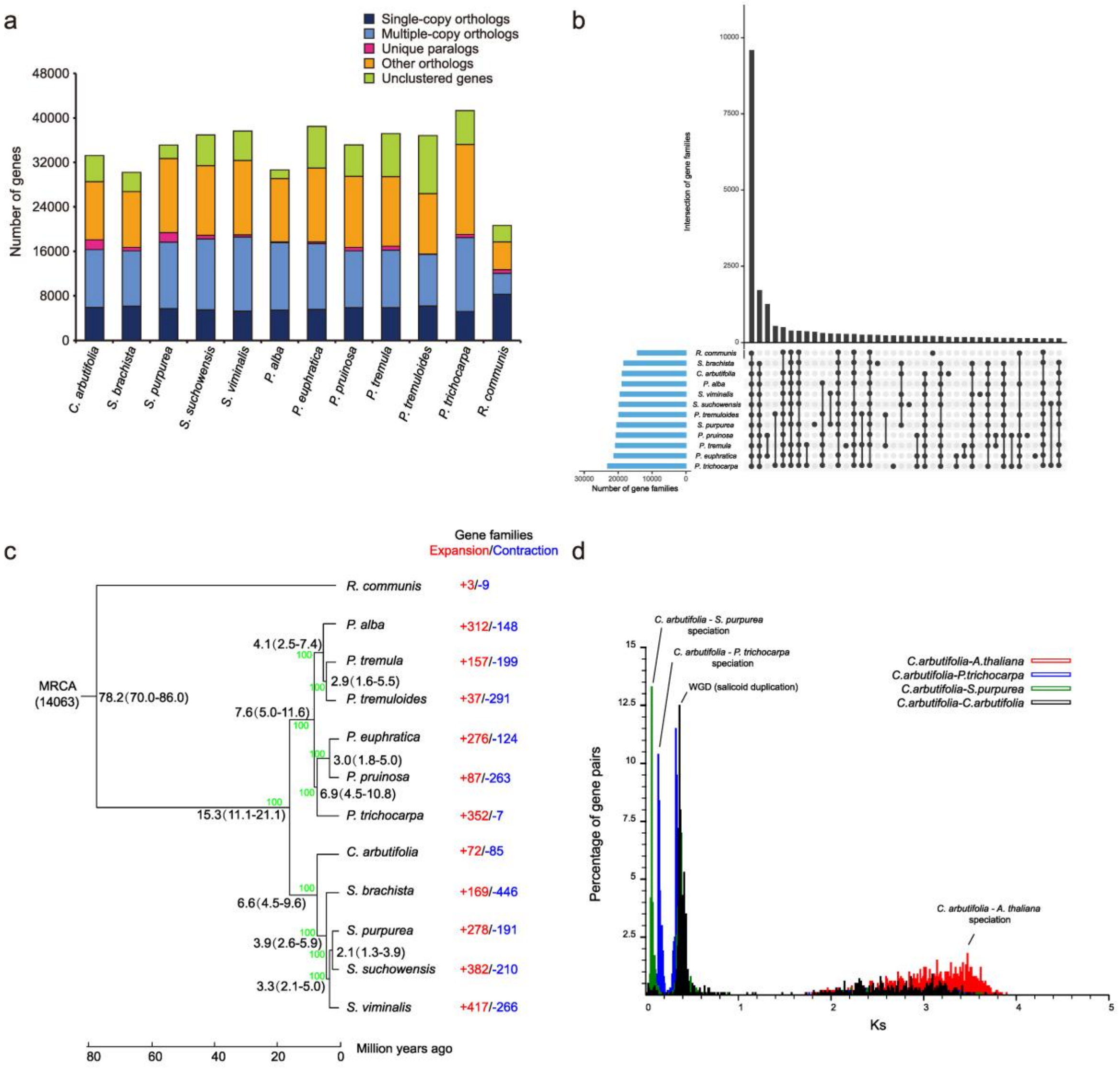

Figure 3: Genome comparison of different Malpighiales species. **a,** Protein orthology comparison in genomes of the indicated 12 species. **b,** Intersections of gene families among the 12 species. Rows and columns represent gene families and intersections, respectively. Black and gray circles indicate the existence or absence of a given intersection. Vertical black lines connecting black circles in each column represent the column based relationship. The bar chart located at the top of the matrix indicate the intersection size. The horizontal bar chart on the left side of the matrix indicates the size of gene family. **c,** Phylogenetic tree of the 12 species. Numbers (black) on nodes indicate the differentiation time, and error ranges are shown in parentheses. **d,** Genome duplication in the *C. arbutifolia* genome revealed by 4DTv analysis.

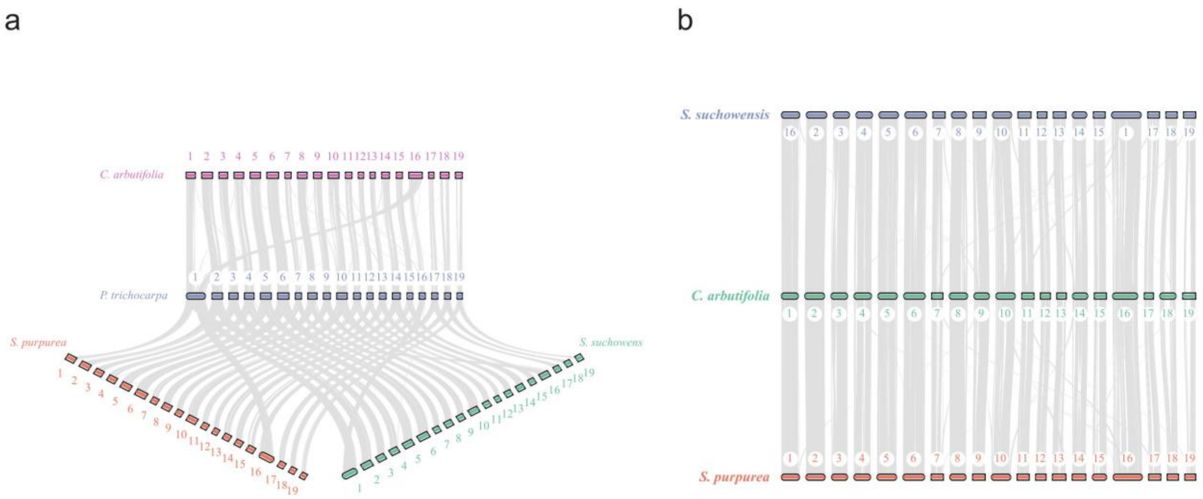

Figure 4: Synteny analysis. **a,** Synteny analysis of *C. arbutifolia*, *S. purpurea*, *S. suchowensis*, and *P. trichocarpa*. **b,** Synteny analysis of *C. arbutifolia*, *S. purpurea*, and *S. suchowensis*. Macrosynteny connecting blocks of >30 one-to-one gene pairs are shown.

Click here to access/download
Supplementary Material
Supplementary tables (resubmission).xlsx
