# Supplementary material for: The whole-genome assembly of an endangered Salicaceae species: Chosenia arbutifolia (Pall.) A. Skv
Source: Gigascience. 2022 Nov 14;11:giac109. doi: 10.1093/gigascience/giac109 (PMC9661892; doi:10.1093/gigascience/giac109)
Supplement: giac109_Response_to_Reviewer_Comments_Original_Submission [file giac109_response_to_reviewer_comments_original_submission.pdf]

Author's Response To Reviewer Comments

Close

Dear Editor,

We sincerely thank you and the reviewers for your valuable comments and instructive advice, which help us to improve and revise our manuscript. We have made detailed changes accordingly. Our point-by-point responses to the reviewers' comments are as follows for your consideration.

Response to Reviewer #1:

He et al. reported the genome assembly and characterization of an endangered Salicaceae species, Chosenia arbutifolia, with significant values in phylogenetic and evolutionary study of the important family. The methods are appropriate, the genome data are generally in high quality, and genome characterization revealed the key findings in Chosenia phylogeny and genome history.

Recommend to accept with minor revision.

Minor Comments:
page 4, line 23, why S. suchowensis was collected? Is it a part of this study reported here?
Answer 1: Sorry for the misleading. In the first place there were some biological analyses in the manuscript. We have modified this part, please see page 5, line 2.

page 5, line 2, "transported back to the laboratory", please be specific, as we have many many labs.
Answer 2: Yes, the institute name was added, please see page 5, line 4.

page 6, line 2-4, please provide full information on genome assembly, the parameters, settings, main considerations.
Answer 3: Yes, the DipAsm software depends on packages of Peregrine, 3d-dna, minimap2, DeepVariant, whatshap and hapcut2. The detailed parameters and settings used for DipAsm assembly were described in Supplementary Table 1. Please see page 6, line 5.

page 6, line 13, I do not think BUSCO could provide assessment on sequence integrity. It could be a kind of assessment on completeness of the genome assembly.
Answer 4: Yes, the word "integrity" was change to "completeness". Please see page 6, line 16.

page 7, line 18, where are the RNA-seq data from? Please provide full information.
Answer 5: The RNA was extracted from the leaves of C. arbutifolia using An Omega Plant RNA Kit (Omega Bio-tek, Norcross, GA, USA). Please see page 5, line 6, the section of "Plant materials and nucleic acid extraction".

page 9, line 16-17, the contig N50 is about 1.68 Mb which is not good as expected. Given the small genome size, the high sequencing depth of HiFi reads, it is necessary to check what happens there, why you just got such a low N50? Could you provide length distribution of your HiFi data? Or any other clues you have?
Answer 6: Yes, the length distribution of HiFi data was shown in figure 1a. The Kmer analysis conducted by GCE (Binghang Liu, et. al. 2013) showed that C. arbutifolia is a highly heterozygous (2.5% heterozygous rate) and highly repetitive (49.17% repeat content) species, which would affect the quality of the assembly, resulting a relatively low N50 (figure 1c).

Reference:
Binghang Liu, Yujian Shi, Jianying Yuan, et al. Estimation of genomic characteristics by analyzing k-mer frequency in de novo genome project. arXiv.org arXiv: 1308.2012. (2013)

page 10, line 3-4, please provide bootstrap values for the tree you presented here.
Answer 7: Yes, the bootstrap values were added, please see Figure 3c.

page 14, line 9, took -> provides or shed?
Answer 8: Yes, the word “took” was changed to “shed”, please see page 15, line 5.

Response to Reviewer #2:

The article is written in the correct language, the methods are applied in accordance with the standards used in plant genomics. On the other hand, it is a pity that the authors did not include Salix dunnii in their phylogenetic studies, even though the genome has been available since December 2020.

Some minor statements need correction:

Page 3 - line 6, statements need clarification - willows (Salix) are also wind pollinated, in particular some apline species exhibit both wind and insect pollination https://doi.org/10.1139/b99-003
Answer 1: Yes, this statement was adjusted, please see page 3, line 6.

Page 14 - line 17, the BioProject number is not found in the NCBI database, is it correct?
Answer 2: The BioProject number was correct, and you can access the data by the link:
https://www.ncbi.nlm.nih.gov/bioproject/PRJNA788330

Response to Reviewer #3:

The manuscript titled "The whole-genome assembly of an endangered Salicaceae species: Chosenia arbutifolia (Pall.) A. Skv." provides a chromosome-scale genome assembly and annotation for C. arbutifolia, a Salix genome of great economic and ecological importance. In addition, by comparing with other genomes, the authors characterized the evolutionary relations between C. arbutifolia and Salicaceae species. Overall, this article provided informative and useful genomic resources for a Chosenia species, but the authors should provide additional details in the use of their methods and descriptions for results which lack several critical details listed below.

Major concerns:
1. Page 7, line 11-12: This paper selected seven plant genomes for homologous gene prediction, but only Populus trichocarpa belongs to Salicaceae. Now that various Populus and Salix genomes have been published and some of them have been applied for gene family analysis, why didn't choose them for gene prediction? How do you evaluate the correctness of this part of result?
Answer 1: Thank you for the good question. We selected seven plant genomes for homologous gene prediction, among which two species (Salix purpurea and Populus trichocarpa) belongs to Salicaceae. As the genus Chosenia is a unique member of the family Salicaceae along with Populus and Salix, the genetic composition of C. arbutifolia may be different from that of other Salicaceae species, so we selected not only Salix and Populus species but also other closely related plants for homology annotation. In addition, the complete BUSCOs of the predicted proteins is about 97.3%, indicating the high quality of the annotated genes, please see Supplementary Table 8. We also added a statement in the text, please page 11, line 19.

2. Page 9, line 23: What is the genomic reason for the difference between C. arbutifolia and other Populus species now that significant size changes were discovered?
Answer 2: The genome size of C. arbutifolia and most diploid Salix species are substantially smaller than Populus species. A repeat-driven genome expansion event was discovered in P. euphratica in early report (Zhang et al. 2020), which may be a possible reasons for the difference in genome size between C. arbutifolia and other Populus species. In addition, some previous studies have reported that Salix species evolved faster than Populus species and might be subjected to stronger purifying selection than poplars, resulting in a reduced number of predicted genes and an overall smaller genome size (Dai et al. 2014; Hou et al. 2019; Wei et al. 2020)

Reference:
Dai et al. The willow genome and divergent evolution from poplar after the common genome duplication. Cell Res. 2014, 24: 1274.
Hou et al. Uneven selection pressure accelerating divergence of Populus and Salix. Horticul Res. 2019, 6:37.
Wei et al. The chromosome-scale assembly of the willow genome provides insight into Salicaceae genome evolution. Horticul Res. 2020, 7:45.
Zhang et al. Improved genome assembly provides new insights into genome evolution in a desert poplar

(Populus euphratica). Mol Ecol Resour. 2020, 20(3): 781-794.

3. Page 10, line 3: This article applied BUSCO for genome assembly evaluation, but obviously this is not enough. How is this genome supported by the sequencing data, such as the mapping rate/data coverage of the NGS/hifi reads? What is the error rate, i.e. the homozygous snp ratio? Besides, what is the alignment result of RNA-seq data? Are there any assembled contigs that may belong to potential bacterial or virual pollution?
Answer 3: Thanks for your valuable suggestions. The NGS reads' mapping rate and coverage are 98.15% and 99.29%, respectively. The HiFi reads' mapping rate and coverage are 93.66% and 95.91%, respectively. The alignment rate of RNA-seq data is 80.81%. The error rate (homozygous snp ratio) is about 0.02%. The distribution of CG depth indicates there is no apparent contamination in the assembled contigs (Figure 1d). In addition, the assembly has been uploaded to NCBI and passed the NCBI Contamination Screen. All these information indicate that the C. arbutifolia assembly is of high quality and have been added to the text. Please see page 10, line 15.

4. Page 12, line 20-22: What is the biological or evolutionary meaning of the PSGs in the C. arbutifolia genome?
Answer 4: As we submitted the manuscript as Date Note article type, which requires little biological analysis, we did not describe the biological meaning of PSGs. Indeed, among these PSGs, one gene associated with "ATP binding" functions as a growth-regulating factor, and another "integral component of membrane" enriched gene is related to calcium-transporting ATPase. The positive selection of these genes may be related to the characteristics that C. arbutifolia is not easy to form adventitious roots.

5. What is the concrete method of genome collinearity analysis? And what is the statistical data of chromosome fusions and fissions between Salicaceae species, such as related gene numbers or chromosome length?
Answer 5: Syntenic regions between Salicaceae assemblies were based on homology searches carried out by using Mcscan-python (https://github.com/tanghaibao/jcvi/wiki/MCscan-(Python-version)) requiring at least 30 genes per block. We identified 2135, 3351 and 2829 genes in the interchromosomal-recombination regions compared with P. trichocarpa for C. arbutifolia, S. purpurea and S. suchowensis, respectively, which may be related with inter-chromosome fusion and fission events. We have added this statement in Method section, and the detailed information could be found in Supplementary Table 15 and Supplementary Table 16.

Minor concerns:

1. Page 7, line 8: How did the neutral mutation rate was selected? Is there any published paper which can support it?
Answer 6: Yes, the neutral mutation rate is referred to by Zhou R, et al., which used a mutation rate of 2.5×10^{-9} to estimate the LTR insertion time for Salix purpurea. We have added this reference in the text. Please see page 7, line 13.

Reference:
Zhou et al. A willow sex chromosome reveals convergent evolution of complex palindromic repeats. Genome Biol. 2020, 21(1):38.

2. Page 9, line 19: Please provide the Hi-C interaction heatmap. In addition, what is the anchoring rate of contig sequences?
Answer 7: The Hi-C interaction heatmap was attached in Figure 2b. A C. arbutifolia genome with a total size of 338.93 Mb was acquired and 95.31% of the assembly sequence was assigned to 19 pseudochromosomes. Please see page 10, line 6.

3. What is the BUSCO result of the predicted protein sequences?
Answer 8: The complete BUSCOs of predicted protein sequences is 97.3%, please see page 11, line 19, and Supplementary Table 8.

4. Please add the estimated genome size for C. arbutifolia if available.
Answer 9: The estimated genome size of C. arbutifolia is 323 Mb. We have added this in the text, please see page 10, line 8.

5. What is the LAI value of the C. arbutifolia genome?
Answer 10: The LTR Assembly Index (LAI) of C. arbutifolia assembly is 10.86. We have added this data in Table 1.

6. Is there any telomere regions, such as 'CCCTAAA' units which have been broadly discovered in plants, were identified? If so, how many chromosomes have telomere regions.
Answer 11: Yes, we have added this analysis using Telomere Identification Toolkit, please see page 10, line 9, and Figure 1e.

7. Page 13, line 2-7: For the WGD event, please provide the result based on the ks distribution.
Answer 12: Yes, we have modified this result using Ks distribution and replaced the relevant figure. Please see page 13, line 19, and Figure 3d.

Close
