# Supplementary material for: The whole-genome assembly of an endangered Salicaceae species: Chosenia arbutifolia (Pall.) A. Skv
Source: Gigascience. 2022 Nov 14;11:giac109. doi: 10.1093/gigascience/giac109 (PMC9661892; doi:10.1093/gigascience/giac109)
Supplement: giac109_Reviewer_1_Report_Original_Submission — Jian-Feng Mao, Ph.D. -- 7/18/2022 Reviewed [file giac109_reviewer_1_report_original_submission.pdf]

Reviewer Report

Title: The whole-genome assembly of an endangered Salicaceae species: Chosenia arbutifolia (Pall.) A. Skv.

Version: Original Submission **Date:** 7/18/2022

Reviewer name: Jian-Feng Mao, Ph.D.

Reviewer Comments to Author:

He et al. reported the genome assembly and characterization of an endangered Salicaceae species, Chosenia arbutifolia, with significant values in phlylogentic and evolutionary study of the important family. The methods are appropriate, the genome data are generally in high quality, and genome characterization revealed the key findings in Chosenia phylogeny and genome history.
Recommend to accept with minor revision.
Minor Comments:
page 4, line 23, why S. suchowensis was collected? Is it a part of this study reported here?
page 5, line 2, "transported back to the laboratory", please be specific, as we have many many labs.
page 6, line 2-4, please provide full information on genome assembly, the parameters, settings, main considerations.
page 6, line 13, I do not think BUSCO could provide assessment on sequence integrity. It could be a kind of assessment on completeness of the genome assembly.
page 7, line 18, where are the RNA-seq data from? Please provide full information.
page 9, line 16-17, the contig N50 is about 1.68 Mb which is not good as expected. Given the small genome size, the high sequencing depth of HiFi reads, it is necessary to check what happens there, why you just got such a low N50? Could you provide length distribution of your HiFi data? Or any other clues you have?
page 10, line 3-4, please provide bootstrap values for the tree you presented here.
page 14, line 9, took -> provides or shed?

Level of Interest

Please indicate how interesting you found the manuscript: Choose an item.

Quality of Written English

Please indicate the quality of language in the manuscript: Choose an item.

Declaration of Competing Interests

Please complete a declaration of competing interests, considering the following questions:

- Have you in the past five years received reimbursements, fees, funding, or salary from an organisation that may in any way gain or lose financially from the publication of this manuscript, either now or in the future?
- Do you hold any stocks or shares in an organisation that may in any way gain or lose financially from the publication of this manuscript, either now or in the future?
- Do you hold or are you currently applying for any patents relating to the content of the manuscript?
- Have you received reimbursements, fees, funding, or salary from an organization that holds or has applied for patents relating to the content of the manuscript?
- Do you have any other financial competing interests?
- Do you have any non-financial competing interests in relation to this paper?

If you can answer no to all of the above, write 'I declare that I have no competing interests' below. If your reply is yes to any, please give details below.

I declare that I have no competing interests

I agree to the open peer review policy of the journal. I understand that my name will be included on my report to the authors and, if the manuscript is accepted for publication, my named report including any attachments I upload will be posted on the website along with the authors' responses. I agree for my report to be made available under an Open Access Creative Commons CC-BY license (http://creativecommons.org/licenses/by/4.0/). I understand that any comments which I do not wish to be included in my named report can be included as confidential comments to the editors, which will not be published.

Choose an item.

To further support our reviewers, we have joined with Publons, where you can gain additional credit to further highlight your hard work (see: https://publons.com/journal/530/gigascience). On publication of this paper, your review will be automatically added to Publons, you can then choose whether or not to claim your Publons credit. I understand this statement.

Yes Choose an item.
