# Supplementary material for: The whole-genome assembly of an endangered Salicaceae species: Chosenia arbutifolia (Pall.) A. Skv
Source: Gigascience. 2022 Nov 14;11:giac109. doi: 10.1093/gigascience/giac109 (PMC9661892; doi:10.1093/gigascience/giac109)
Supplement: giac109_Reviewer_3_Report_Original_Submission — Tao Ma -- 8/8/2022 Reviewed [file giac109_reviewer_3_report_original_submission.pdf]

Reviewer Report

Title: The whole-genome assembly of an endangered Salicaceae species: Chosenia arbutifolia (Pall.) A. Skv.

Version: Original Submission **Date:** 8/8/2022

Reviewer name: Tao Ma

Reviewer Comments to Author:

The manuscript titled "The whole-genome assembly of an endangered Salicaceae species: Chosenia arbutifolia (Pall.) A. Skv." provides a chromosome-scale genome assembly and annotation for C. arbutifolia, a Salix genome of great economic and ecological importance. In addition, by comparing with other genomes, the authors characterized the evolutionary relations between C. arbutifolia and Salicaceae species. Overall, this article provided informative and useful genomic resources for a Chosenia species, but the authors should provide additional details in the use of their methods and descriptions for results which lack several critical details listed below.

Major concerns:

1. Page 7, line 11-12: This paper selected seven plant genomes for homologous gene prediction, but only Populus Trichocarpa belongs to Salicaceae. Now that various Populus and Salix genomes have been published and some of them have been applied for gene family analysis, why didn't choose them for gene prediction? How do you evaluate the correctness of this part of result?
2. Page 9, line 23: What is the genomic reason for the difference between C. arbutifolia and other Populus species now that significant size changes were discovered?
3. Page 10, line 3: This article applied BUSCO for genome assembly evaluation, but obviously this is not enough. How is this genome supported by the sequencing data, such as the mapping rate/data coverage of the NGS/hifi reads? What is the error rate, i.e. the homozygous snp ratio? Besides, what is the alignment result of RNA-seq data? Are there any assembled contigs that may belong to potential bacterial or virual pollution?
4. Page 12, line 20-22: What is the biological or evolutionary meaning of the PSGs in the C. arbutifolia genome?
5. What is the concrete method of genome collinearity analysis? And what is the statistical data of chromosome fusions and fissions between Salicaceae species, such as related gene numbers or chromosome length?

Minor concerns:

1. Page 7, line 8: How did the neutral mutation rate was selected? Is there any published paper which can support it?
2. Page 9, line 19: Please provide the Hi-C interaction heatmap. In addition, what is the anchoring rate of contig sequences?
3. What is the BUSCO result of the predicted protein sequences?
4. Please add the estimated genome size for C. arbutifolia if available.
5. What is the LAI value of the C. arbutifolia genome?

6. Is there any telomere regions, such as 'CCCTAAA' units which have been broadly discovered in plants, were identified? If so, how many chromosomes have telomere regions.
7. Page 13, line 2-7: For the WGD event, please provide the result based on the ks distribution.

Level of Interest

Please indicate how interesting you found the manuscript: Choose an item.

Quality of Written English

Please indicate the quality of language in the manuscript: Choose an item.

Declaration of Competing Interests

Please complete a declaration of competing interests, considering the following questions:

- Have you in the past five years received reimbursements, fees, funding, or salary from an organisation that may in any way gain or lose financially from the publication of this manuscript, either now or in the future?
- Do you hold any stocks or shares in an organisation that may in any way gain or lose financially from the publication of this manuscript, either now or in the future?
- Do you hold or are you currently applying for any patents relating to the content of the manuscript?
- Have you received reimbursements, fees, funding, or salary from an organization that holds or has applied for patents relating to the content of the manuscript?
- Do you have any other financial competing interests?
- Do you have any non-financial competing interests in relation to this paper?

If you can answer no to all of the above, write 'I declare that I have no competing interests' below. If your reply is yes to any, please give details below.

I declare that I have no competing interests

I agree to the open peer review policy of the journal. I understand that my name will be included on my report to the authors and, if the manuscript is accepted for publication, my named report including any attachments I upload will be posted on the website along with the authors' responses. I agree for my report to be made available under an Open Access Creative Commons CC-BY license (http://creativecommons.org/licenses/by/4.0/). I understand that any comments which I do not wish to be included in my named report can be included as confidential comments to the editors, which will not be published.

Choose an item.

To further support our reviewers, we have joined with Publons, where you can gain additional credit to further highlight your hard work (see: https://publons.com/journal/530/gigascience). On publication of this paper, your review will be automatically added to Publons, you can then choose whether or not to claim your Publons credit. I understand this statement.

Yes Choose an item.
